# Safety and Efficacy Analysis of Selinexor-Based Treatment in Multiple Myeloma, a Meta-Analysis Based on Prospective Clinical Trials

**DOI:** 10.3389/fphar.2021.758992

**Published:** 2021-12-03

**Authors:** Yali Tao, Hui Zhou, Ting Niu

**Affiliations:** Department of Hematology and Research Laboratory of Hematology, West China Hospital, Sichuan University, Chengdu, China

**Keywords:** selinexor, multiple myeloma, XPO1, efficacy, safety, meta-analysis

## Abstract

**Background:** Selinexor (SEL) is an orally bioavailable, highly-selective, and slowly-reversible small molecule that inhibits Exportin 1. Preclinical studies showed that SEL had synergistic antimyeloma activity with glucocorticoids, proteasome inhibitors (PIs) and immunomodulators. The combination of selinexor and dexamethasone (DEX) has been approved in the United States for patients with penta-refractory multiple myeloma in July 2019. This meta-analysis aimed to investigate the safety and efficacy of selinexor based treatment in Multiple myeloma.

**Methods:** We systematically searched the Medline (PubMed), Embase, Web of Science, Cochrane Central Register of Controlled Trials Library databases and ClinicalTrials.gov. Outcome measures of efficacy included overall response rate (ORR), clinical benefit rate (CBR), stringent complete response rate (sCR), complete response rate (CR), very good partial response (VGPR), partial response rate (PR), minimal response (MR), rate of stable disease (SDR), rate of progressive disease (PDR) and median progression-free survival (mPFS). Safety was evaluated by the incidences of all grade adverse events and Grade≥3 adverse events. The subgroup analysis was conducted to analyze the difference in different combination treatment regimens (SEL + DEX + PIs vs SEL + DEX).

**Results:** We included six studies with 477 patients. The pooled ORR, CBR, sCR, CR, VGPR, PR, MR, SDR, and PDR were 43% (18–67%), 55% (32–78%), 5% (−2–13%), 7% (4–11%), 14% (5–24%), 23% (15–31%), 11% (8–14%), 26% (14–38%) and 14% (4–23%), respectively. SEL + DEX + PIs treatment had higher ORR (54 vs 24%, *p* = 0.01), CBR (66 vs 37%, *p* = 0.01), sCR (10 vs 2%, *p* = 0.0008), and VGPR (23 vs 5%, *p* < 0.00001) compared to SEL + DEX treatment, and lower PDR (4 vs 23%, *p* < 0.00001) and SDR (17 vs 37%, *p* = 0.0006). The pooled incidences of any grade and grade≥3 were 45 and 30% in hematological AEs, and in non-hematological AEs were 40 and 30%, respectively. The most common all grade (68%) and grade≥3 (54%) hematological AE were both thrombocytopenia. Fatigue was the most common all grade (62%) and grade≥3 (16%) non-hematological AE. Compared to SEL + DEX treatment, SEL + DEX + PIs treatment had lower incidences of hyponatremia (39 vs 12%, *p* < 0.00001), nausea (72 vs 52%, *p* < 0.00001), vomiting (41 vs 23%, *p* < 0.0001), and weight loss (42 vs 17%, *p* = 0.03) in all grade AEs. Meanwhile, SEL + DEX + PIs treatment had lower incidences of anemia (36 vs 16%, *p* = 0.02), fatigue (20 vs 13%, *p* = 0.04), hyponatremia (22 vs 5%, *p* < 0.0001) than SEL + DEX treatment in grade≥3 AEs.

**Conclusion:** Our meta-analysis revealed that selinexor-based regimens could offer reasonable efficacy and tolerable adverse events in patients with multiple myeloma. SEL + DEX + PIs treatments had higher efficacy and lower toxicities than SEL + DEX.

## Introduction

Multiple myeloma (MM) is a bone marrow-based malignant plasma-cell disorder that is characterized by production of monoclonal immunoglobulin (M protein), osteolytic bone lesions, hypercalcemia, anemia and associated organ dysfunction (Palumbo and Anderson, 2011). Over the past 2 decades, survival of patients with multiple myeloma has significantly improved as a result of the introduction of autologous stem cell transplantation (ASCT) and several novel classes of drugs, including proteasome inhibitors (PIs) (bortezomib, carfilzomib and ixazomib), immunomodulatory agents (IMiDs) (thalidomide, lenalidomide and pomalidomide), monoclonal antibodies targeting CD38 (daratumumab, isatuximab) and SLAMF7 (elotuzumab), and HDAC inhibitors (panobinostat) (Miguel et al., 2013; Dimopoulos et al., 2015; Lonial et al., 2015; Dimopoulos et al., 2016; San-Miguel et al., 2016; Chim et al., 2018; Goldschmidt et al., 2019; Kumar et al., 2019). However, none of these agents are curative, despite these additions to the MM armamentarium, most patients will relapse and develop refractory disease to all available therapies (Kumar et al., 2019; Kumar et al., 2012; Gandhi et al., 2019). Therefore, it remains a high priority to develop novel, more efficacious and less toxic treatment strategies for patients with relapsed/refractory MM.

Exportin 1 (XPO1), the only known nuclear export protein for most tumor suppressor proteins, cell-cycle regulators, the glucocorticoid receptor, and several eIF4A-bound oncoprotein mRNAs for key oncoproteins, including c-MYC, BCL-2 and Cyclin D (Gaubatz et al., 2001; Culjkovic-Kraljacic et al., 2012; Gravina et al., 2014). XPO1 is overexpressed in multiple myeloma and correlates with increased bone disease and shorter survival (Tiedemann et al., 2012; Tai et al., 2014), a genome-wide RNA interference screen identified XPO1 as an essential gene required for myeloma cell survival and proliferation (Schmidt et al., 2013). Furthermore, elevated levels of XPO1 is associated with the development of resistance to proteasome inhibitors (including bortezomib) (Chanukuppa et al., 2019) and immunomodulatory agents (Bhutani et al., 2017).

Selinexor (SEL) is an orally bioavailable, highly-selective, and slowly-reversible small molecule that binds to the Cys528 residue in the cargo-binding pocket of XPO1 (Gravina et al., 2014). Treatment of cancer cells with selinexor induced nuclear retention of tumor suppressor proteins (TSPs) and blocked the export of eIF4E-bound oncoprotein mRNAs, leading apoptosis, reduced levels of proto-oncoproteins and impaired osteoclastogenesis (Gravina et al., 2014; Tan et al., 2014). Preclinical studies have shown that selective inhibitor of nuclear export compounds showed noticeable anti-myeloma activity largely independent of genotype, as well as synergistic activity with glucocorticoids, proteasome inhibitors, and immunomodulatory drugs (Tai et al., 2014; Turner et al., 2014; Turner et al., 2016a). The combination of selinexor (80 mg, twice per week) and dexamethasone (DEX) has been approved in the United States for patients with penta-refractory multiple myeloma in July 2019 based on the STORM clinical trial. There are several prospective clinical trials having been conducted to investigate the safety and efficacy of selinexor-based treatment in patients with MM. However, a quantitative and comprehensive meta-analysis focus on the safety and efficacy of selinexor-based treatment is still scarce. In this study, we analyzed the comprehensive safety and efficacy of selinexor-based treatments in patients with relapsed or/and refractory MM, and overcome the limitations of individual studies, such as small sample size and lack of statistical power. Besides, we also used subgroup analysis to compare the efficacy and safety of different combination therapies (SEL + DEX + PIs vs SEL + DEX). These findings lead to the evaluation of XPO1 inhibitors for the treatment of MM.

## Methods

### Study Design, Search Strategy

This systematic review and meta-analysis was conducted in compliance with the Preferred Reporting Items for Systematic Reviews and Meta-Analyses (PRISMA) statement. We searched the Medline (PubMed), Embase, Web of Science, Cochrane Central Register of Controlled Trials Library databases and ClinicalTrials.gov by a combination of Medical Subject Heading terms and keywords regarding “selinexor” and “multiple myeloma” to identify studies assessing selinexor based treatment in the setting of multiple myeloma. There were no date or language restrictions and the data cut-off for this analysis was March 5, 2021. The detailed search strategy was provided in Supplementary Materials.

### Inclusion Criteria and Excluded Criteria

Studies that met the following criterias were included: 1) clinical trials in any phase of selinexor-based therapy for patients with multiple myeloma; 2) full data of the safety and efficacy were available in the articles; 3) the drugs were applied on human; 5) patients’ age was over 18 years.

The studies were excluded if they met the following criterias: 1) the studies were without initial data such as reviews; 2) it was a case report. When more than one study reported the results from the same cohort, only the most recent study was included.

Two authors independently searched, screened, and determined study eligibility, with any disagreement were resolved by discussion.

### Data extraction

Two investigators independently reviewed and extracted the following Data: 1) the fundamental characteristics of included studies: the first author name, year of publication, ClinicalTrials.gov number, the phase of the studies, number of participating patients, prior lines of treatment, median age, intervention; 2) the AEs of all grades and grade ≥3 AEs; 3) efficacy outcome such as overall response rate (ORR), clinical benefit rate (CBR), stringent complete response rate (sCR), complete response rate (CR), very good partial response rate (VGPR), partial response rate (PR), minimal response rate (MR), rate of stable disease (SDR), rate of progressive disease (PDR) and median progression-free survival (mPFS). Discrepancies were settled by discussion. For the RCT, we only extracted the information of selinexor-based treatment.

### Statistical Analysis

The Revman 5.4 and STATA14 (Stata Corporation) were used in this study. The I -squared test ( $I^{2}$ test) was used to evaluate between-study heterogeneity. The $I^{2}$ statistic ranges from 0 to 100% ( $I^{2}$ <25%, low heterogeneity. $I^{2}$ 25–50%, moderate heterogeneity. $I^{2}$ >50%, significant heterogeneity). A random effects model was used when $I^{2}$ >50%, the fixed-effect model was employed when $I^{2}$ ≤50%. Galbraith plots were used to explore potential sources of heterogeneity (Higgins et al., 2003). All analyses were based on the intention-to-treat population of the included studies. The subgroup analysis by treatment (SEL + DEX + PIs vs SEL + DEX) was conducted to analyze the differences among different combination treatment regimens.

### Study Qualitative Assessment

We used the Cochrane Collaboration Risk of Bias Tool (Higgins et al., 2011) (Review Manager 5.4) to assess systematic bias of the involved RCT study, which is based on six criteria: sequence generation, allocation concealment, blinding of participants, blinding of outcomes, completeness of the data, and selective outcome reporting. The Methodological Index for Non-randomized Studies (MINORS) was adopted to assess the methodological quality of the inclusive non-RCT studies. MINORS contained 12 items, eight of which were specified for non-comparative studies (Slim et al., 2003). The eight items included: study aims, consecutive patient inclusion criteria, prospective pooling of data, endpoint consistent with the study aim, unbiased evaluation of endpoints, follow-up period, loss to follow-up less than 5%, and prospective calculation of the sample size. The items were scored 0 (not reported), 1 (reported but inadequate), or 2 (reported and adequate). Two reviewers independently evaluated the quality of each study, and the discrepancy was solved by consensus.

## Results

### Study Selection and Characteristics

**FIGURE 1:**
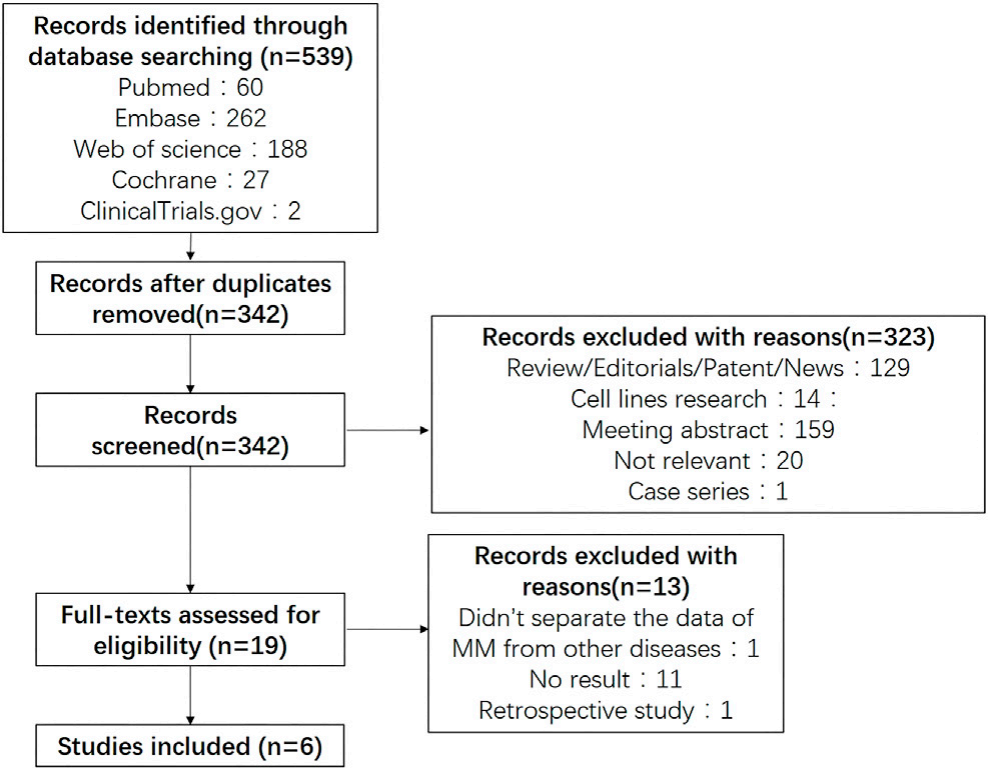
Flow diagram showing the search results.

Five hundred and thirty nine potentially relevant studies were identified through searching PubMed, Embase, Web of Science and CENTRAIL. 197 were removed after duplication, 533 were finally excluded due to different reasons. Figure 1 presented the details of our search process. Six studies (26–31) containing 1 phase III trials and 5 phase I/II trials were selected in our meta-analysis.

The characteristics of the including articles was listed in Table 1. A total of 477 patients were involved in the six studies with the mean age<70 years, among which 4 studies containing 276 patients using SEL + DEX + PIs treatment (Bahlis et al., 2018; Jakubowiak et al., 2019; Grosicki et al., 2020; Salcedo et al., 2020), two studies containing 201 patients using SEL + DEX treatment (Vogl et al., 2018; Chari et al., 2019). The prior lines of therapy patients received ranged from 1 to 18. The selected studies were published from 2018 to 2020. All studies presented complicate information on AEs and response rate. Five studies with 459 patients reported the median progression-free survival. All studies were open-label clinical trials, five were single-arm, one was RCT.

**TABLE 1:** Study characteristics.

Study	Regestion number	Design	Treatment	No. of patients	Age range	Male/female (n)	Previous therapy	NO. of patients received ASCT,n (%)	mPFS (m)	MINORs scores	Ref
Andrzej J. Jakubowiak-2019	NCT02199665	Phase I, single arm	SEL + DEX + CFZ	21	55–74	11/10	2–10	20 (95%)	3.7	12	Jakubowiak et al. (2019)
Bahlis, N. J.-2019	NCT02343042	Phase Ib/II, single arm	SEL + DEX + BTZ	42	43–75	23/19	1–11	30 (71%)	9.0	14	Bahlis et al. (2018)
Grosicki, S.-2020	NCT03110562	Phase III, randomized study	SEL + DEX + BTZ	195	59–72	115/80	1–3	76 (39%)	13.93	RCT	Grosicki et al. (2020)
Meghan Salcedo. -2020	—	Phase I, single arm	SEL + DEX + IXZ	18	44–78	10/8	1–11	15 (83%)	-	10	Salcedo et al. (2020)
Chari A.-2019	NCT02336815	Phase IIb, single arm	SEL + DEX	122	40–86	71/51	3–18	102 (84%)	3.7	16	Chari et al. (2019)
Dan T. Vogl-2018	NCT02336815	Phase II, single arm	SEL + DEX	79	34–78	37/42	3–17	61 (77%)	2.3	16	Vogl et al. (2018)

BTZ, bortezomib; CFZ, carfilzomib; DEX, dexamethasone; IXZ, ixazomib; RCT, randomized controlled trail; SEL, Selinexor.

### Assessment of Study Quality

All studies were open-label. The Cochrane Collaboration risk of bias tool was used to assess the methodological quality of the RCT (30), the result of quality evaluation was good. Supplementary Figure S1 shows the risk of bias graph and risk of bias summary of the RCT study. The MINORS scores of other studies ranged from 10 to 16 (Table 1). All studies stated the aim of their study clearly, included of consecutive patients, collected data prospectively and had appropriate endpoints for the aim of the study. Three studies did not report the unbiased assessment of the study endpoint. One study did not report the follow-up period appropriate to the aim of the study. All studies, the patients’ loss to follow-up did not exceed 5%. Two studies did not prospectively calculate the study size. All in all, the overall quality of the selected studies was adequate.

### Publication Bias

According to the results of I -squared test and galbraith plots, there is heterogeneity in the outcomes of ORR (Supplementary Figure S28), CBR (Supplementary Figure S29), VGPR (Supplementary Figure S30), PR (Supplementary Figure S31), SDR (Supplementary Figure S33), and PDR (Supplementary Figure S34), and then pooled outcomes were calculated using random-effects meta-analysis. There is a low risk of heterogeneity in MR (Supplementary Figure S32), and fixed-effect meta-analysis was used for combining data of MR. The assessment of CR and sCR is not possible because of the insufficient number of included studies.

### Efficacy

All study reported the efficacy outcome such as overall response rate (ORR), clinical benefit rate (CBR), stringent complete response rate (sCR), complete response rate (CR), very good partial response (VGPR), partial response rate (PR), minimal response (MR), rate of stable disease (SDR), rate of progressive disease (PDR), and the responses were evaluated using International Myeloma Working Group (IMWG) response criteria.

**FIGURE 2:**
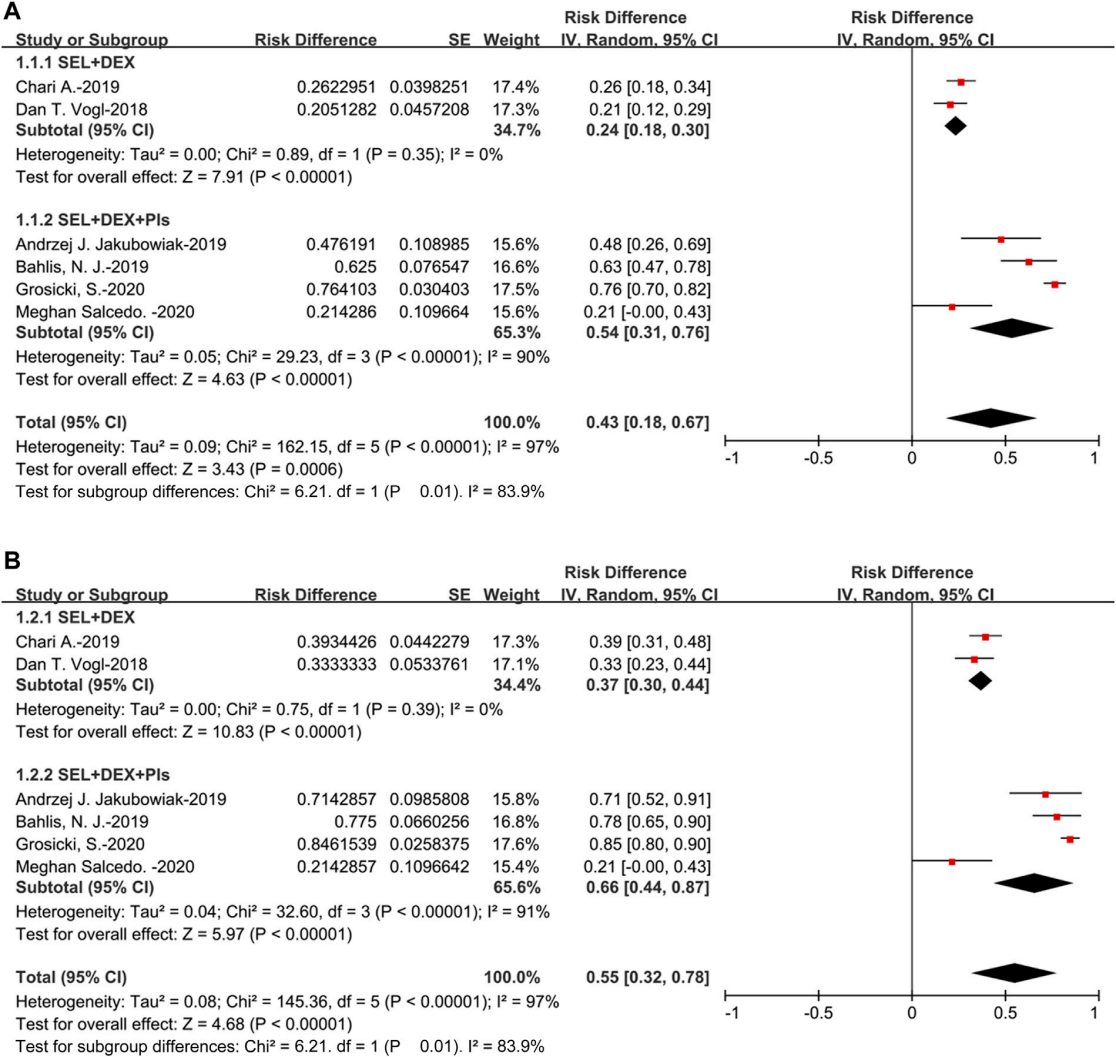
The forest plot of pooled **(A)** ORR, and **(B)** CBR.

All the studies could be used to evaluate ORR, the pooled ORR was 43% (95%CI: 18–67%), suggested that approximately 43% of the 477 patients displayed PR or better as the best response to selinexor-based regimens. For the SEL + DEX + PIs treatment, the pooled ORR was 54% (95%CI: 31–76%), for the SEL + DEX treatment, the ORR was 24% (95%CI: 18–30%). The results of subanalysis showed that the ORR of SEL + DEX treatment was much lower than SEL + DEX + PIs treatment (24 vs 54%, *p* = 0.01) (Figure 2).

Additionally, the pooled CBR was 55% (95%CI: 32–78%), suggested that approximately 55% of the 477 patients displayed MR or better as the best response to selinexor-based regimens. For the subanalysis of SEL + DEX + PIs treatment, the pooled CBR was 66% (95%CI: 44–87%), for the SEL + DEX treatment, the CBR was 37% (95%CI: 30–44%). Subgroup analysis of CBR by treatment showed that, in patients using SEL + DEX + PIs treatment, the CBR was higher than in those using SEL + DEX treatment (66 vs 37%, *p* = 0.01) (Figure 2).

**FIGURE 3:**
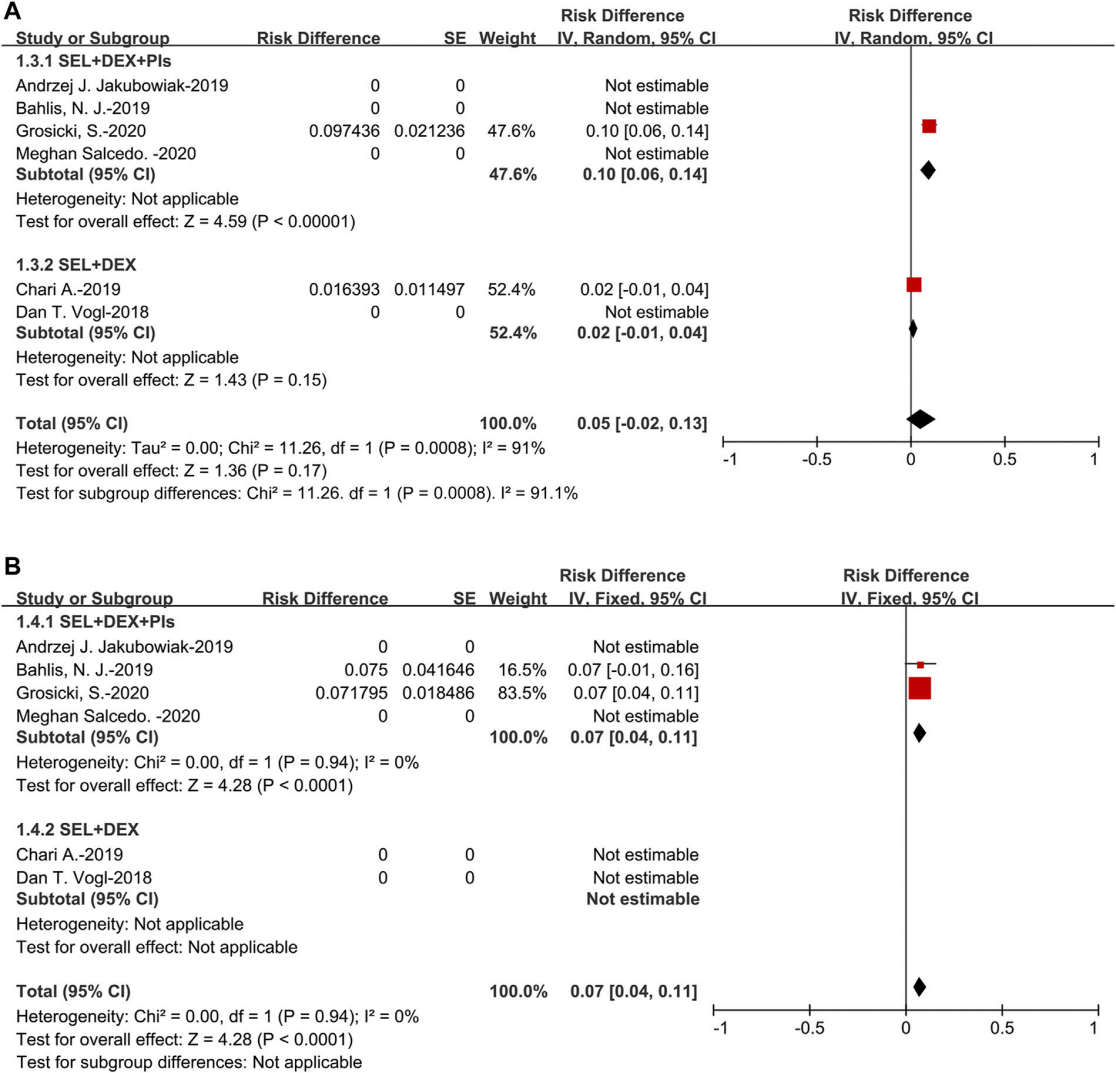
The forest plot of pooled **(A)** sCR, and **(B)** CR.

The pooled sCR was 5% (95%CI: 2–13%). For the subanalysis of SEL + DEX + PIs treatment, the pooled sCR was 10% (95%CI: 6–14%), for the SEL + DEX treatment, the sCR was 2% (95%CI: 1–4%). The pooled sCR of patients using SEL + DEX + PIs treatment was higher than in those using SEL + DEX treatment (10 vs 2%, *p* = 0.0008) (Figure 3).

The pooled CR was 7% (95%CI: 4–11%) (Figure 3). Of the four studies that adopted the SEL + DEX + PIs treatment, two had a CR of 0%, and the other two had CRs of 8% (Bahlis et al., 2018) and 7% (Grosicki et al., 2020), respectively. Both studies that used SEL + DEX treatment (29, 31) had a CR of 0%. Therefore, the subgroup analysis by treatment (SEL + DEX + PIs vs SEL + DEX) was not conducted.

**FIGURE 4:**
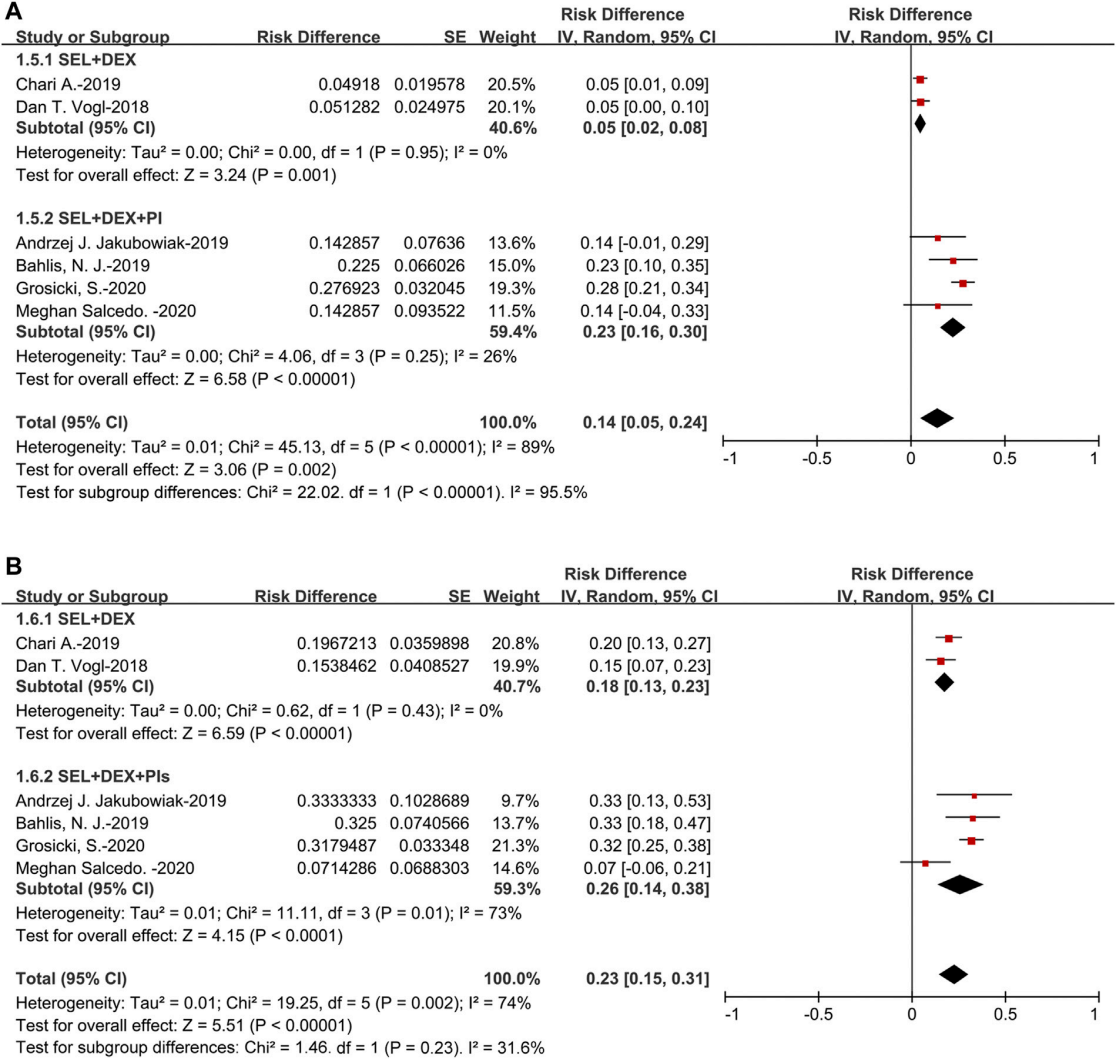
The forest plot of pooled **(A)** VGPR, and **(B)** PR.

The pooled VGPR was 14% (95%CI: 5–24%). For the subanalysis of SEL + DEX + PIs treatment, the pooled VGPR was 23% (95%CI: 16–30%), for the SEL + DEX treatment, the VGPR was 5% (95%CI: 2–8%). The pooled VGPR of patients using SEL + DEX + PIs treatment was higher than in those using SEL + DEX treatment (23 vs 5%, *p* < 0.00001) (Figure 4).

The pooled PR was 23% (95%CI: 15–31%). For the subanalysis of SEL + DEX + PIs treatment, the pooled PR was 26% (95%CI: 14–38%), for the SEL + DEX treatment, the PR was 18% (95%CI: 13–23%). The results of subanalysis indicated that no significant difference between the PR of patients using SEL + DEX + PIs treatment and in those using SEL + DEX treatment (26 vs 18%, *p* = 0.23) (Figure 4).

**FIGURE 5:**
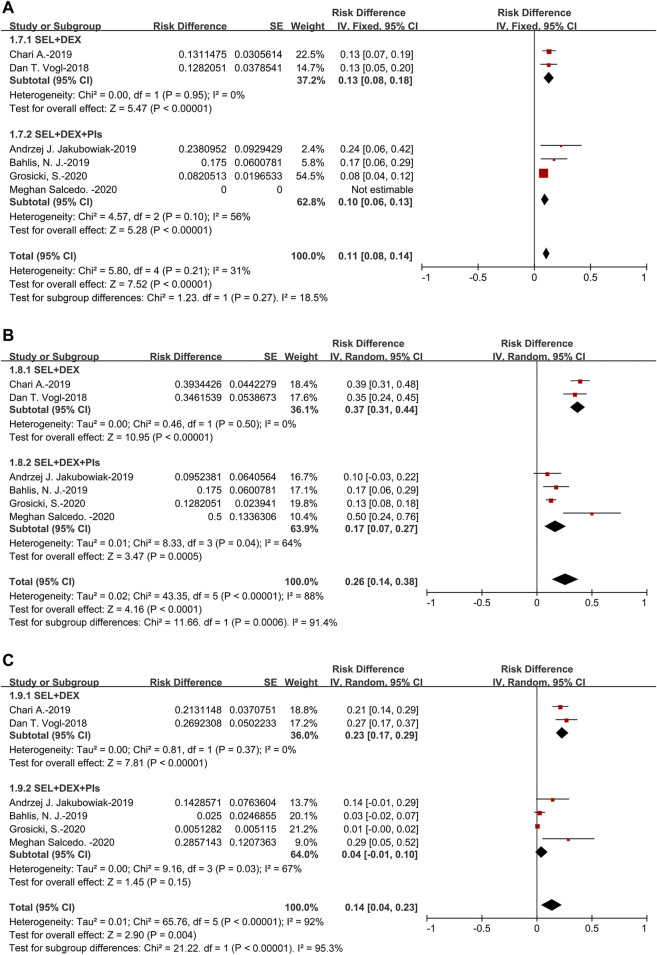
The forest plot of pooled **(A)** MR **(B)** SDR, and **(C)** PDR.

The pooled MR was 11% (95%CI: 8–14%). For the subanalysis of SEL + DEX + PIs treatment, the pooled MR was 10% (95%CI: 6–13%), for the SEL + DEX treatment, the MR was 13% (95%CI: 8–18%). Subgroup analysis of MR suggested that no significant difference existed in the patients using SEL + DEX + PIs treatment and those using SEL + DEX treatment (10 vs 13%, *p* = 0.27) (Figure 5).

The pooled SDR was 26% (95%CI: 14–38%). For the subanalysis of SEL + DEX + PIs treatment, the pooled SDR was 17% (95%CI: 7–27%), for the SEL + DEX treatment, the SDR was 37% (95%CI: 31–44%). The results of subanalysis indicated that the SDR of patients using SEL + DEX treatment was higher than in those using SEL + DEX + PIs treatment (37 vs 17%, *p* = 0.0006) (Figure 5).

The pooled PDR was 14% (95%CI: 4–23%). For the subanalysis of SEL + DEX + PIs treatment, the pooled PDR was 4% (95%CI: 1–10%), for the SEL + DEX treatment, the PDR was 23% (95%CI: 17–29%). The results of subanalysis showed that the PDR of patients using SEL + DEX treatment was higher than in those using SEL + DEX + PIs treatment (23 vs 4%, *p* < 0.00001), which indicated that using SEL + DEX + PIs treatment led to lower disease progression rate than using SEL + DEX treatment (Figure 5). All these results indicated that the efficacy of SEL + DEX + PIs treatment was better than SEL + DEX treatment.

Five studies presented mPFS and the survival curve, with one study (Salcedo et al., 2020) using SEL + DEX + PIs treatment did not present mPFS or the survival curve. For patients using SEL + DEX + PIs treatment, the mPFS in the reported three studies was 3.7, 9.0, and 13.93 months, respectively. For patients using SEL + DEX treatment, the mPFS in the reported two studies was 3.7 and 2.3 months, respectively. It seemed that SEL + DEX + PIs treatment was related to better survival than SEL + DEX treatment. The mPFS recorded in articles on patients were listed in Table1.

### Safety

All the involved articles including 477patients reported AEs, so all studies were contributing to the meta-analysis of the all grade AEs rate and the grade≥3 AEs rate. All treatment-emergent AEs in included studies were graded according to the National Cancer Institute-Common Terminology Criteria for Adverse Events. The pooled incidence of all grade hematological AEs was 45%, and the all grade non-hematological AEs was 40%. The most common all grade hematological AE was thrombocytopenia 68%, and the most common all grade non-hematological was fatigue 62%. The pooled incidence of grade≥3 hematological AEs was 30%, and the grade≥3 non-hematological AEs was 30%. The most common grade≥3 hematological AE was thrombocytopenia 54%, and the most common grade≥3 non-hematological was fatigue 16%. Table 2 listed the pooled incidences of treatment-emergent adverse events.

**TABLE 2:** Pooled incidence of treatment-emergent adverse events.

AEs	Treatment	All grades			Grades≥3		
		Included study	Pooled rate (95%Cl)	*p*-value	Included study	Pooled rate (95%Cl)	*p*-value
Hematological	SEL + DEX + PIs	4	35%, 95%CI (28%,42%)	—	4	0.31%,95%CI (0.22%,40%)	—
	SEL + DEX	2	49%, 95%CI (41%,58%)		2	28%, 95%CI (18%,39%)	
	Overall	6	45%, 95%CI (36%,45%)		6	30%, 95%CI (23%,36%)	
Thrombocytopenia	SEL + DEX + PIs	4	64%, 95%CI (53%,76%)	0.19	4	52%, 95%CI (38%,67%)	0.41
	SEL + DEX	2	73%, 95%CI (67%,79%)		2	59%, 95%CI (52%,66%)	
	Overall	6	68%, 95%CI (60%,76%)		6	54%, 95%CI (44%,65%)	
Anemia	SEL + DEX + PIs	4	45%, 95%CI (26%,65%)	0.31	4	16%, 95%CI (11%,21%)	**0.02**
	SEL + DEX	2	59%, 95%CI (41%,77%)		2	36%, 95%CI (20%,52%)	
	Overall	6	50%, 95%CI (34%,66%)		6	25%, 95%CI (14%,36%)	
Lymphopenia	SEL + DEX + PIs	1	52%, 95%CI (31%,74%)	—	1	33%, 95%CI (13%,53%)	—
	SEL + DEX	2	18%, 95%CI (12%,23%)		2	11%, 95%CI (7%,16%)	
	Overall	3	25%, 95%CI (12%,39%)		3	14%, 95%CI (6%,21%)	
Neutropenia	SEL + DEX + PIs	4	24%, 95%CI (13%,34%)	0.07	4	21%, 95%CI (8%,34%)	0.93
	SEL + DEX	2	36%, 95%CI (28%,44%)		2	22%, 95%CI (16%,27%)	
	Overall	6	29%, 95%CI (18%,40%)		6	21%, 95%CI (13%,29%)	
Leukopenia	SEL + DEX + PIs	0	—	—	0	—	—
	SEL + DEX	2	35%, 95%CI (29%,42%)		2	14%, 95%CI (9%,19%)	
	Overall	2	35%, 95%CI (29%,42%)		2	14%, 95%CI (9%,19%)	
Non-hematological	SEL + DEX + PIs		35%, 95%CI (28%,42%)	-		5%, 95%CI (3%,7%)	-
	SEL + DEX		49%, 95%CI (41%,58%)			8%, 95%CI (5%,11%)	
	Overall		40%, 95%CI (34%,47%)			30%, 95%CI (23%,36%)	
Fatigue	SEL + DEX + PIs	4	59%, 95%CI (41%,77%)	0.34	4	13%, 95%CI (9%,17%)	**0.04**
	SEL + DEX	2	69%, 95%CI (59%,79%)		2	20%, 95%CI (10%,30%)	
	Overall	6	62%, 95%CI (49%,76%)		6	16%, 95%CI (12%,20%)	
Nausea	SEL + DEX + PIs	4	52%, 95%CI (46%,58%)	**<0.00001**	3	7%, 95%CI (4%,10%)	0.52
	SEL + DEX	2	72%, 95%CI (66%,78)		2	9%, 95%CI (5%,13%)	
	Overall	6	61%, 95%CI (51%,72%)		5	8%, 95%CI (5%,10%)	
Decreased appetite	SEL + DEX + PIs	3	33%, 95%CI (6%,60%)	0.14	2	3%, 95%CI (1%,6%)	0.86
	SEL + DEX	2	53%, 95%CI (47%,60%)		2	4%, 95%CI (1%,6%)	
	Overall	5	41%, 95%CI (22%,60%)		4	3%, 95%CI (2%,5%)	
Hyponatremia	SEL + DEX + PIs	3	12%, 95%CI (4%,21%)	**<0.00001**	2	5%, 95%CI (−0%,10%)	***p* < 0.0001**
	SEL + DEX	2	39%, 95%CI (32%,45%)		2	22%, 95%CI (16%,27%)	
	Overall	5	25%, 95%CI (10%,40%)		4	13%, 95%CI (3%,23%)	
Dyspnoea	SEL + DEX + PIs	2	29%, 95%CI (−13%,72%)	—	2	1%, 95%CI (-0%,2%)	—
	SEL + DEX	1	22%, 95%CI (15%,29%		1	4%, 95%CI (1%,8%)	
	Overall	3	24%, 95%CI (8%,40%)		3	2%, 95%CI (-1%,5%)	
Diarrhea	SEL + DEX + PIs	4	35%, 95%CI (27%,44%)	0.09	2	6%, 95%CI (3%,9%)	0.91
	SEL + DEX	2	45%, 95%CI (38%,51%)		3	6%, 95%CI (3%,10%)	
	Overall	6	39%, 95%CI (32%,46%)		5	6%, 95%CI (4%,9%)	
Vomiting	SEL + DEX + PIs	4	23%, 95%CI (18%,28%)	**<0.0001**	3	4%, 95%CI (1%,66%)	0.82
	SEL + DEX	2	41%, 95%CI (34%,47%)		2	3%, 95%CI (1%,6%)	
	Overall	6	32%, 95%CI (22%,41%)		5	4%, 95%CI (2%,5%)	
weight loss	SEL + DEX + PIs	3	17%, 95%CI (3%,31%)	**0.03**	1	2%, 95%CI (0%,4%)	—
	SEL + DEX	2	42%, 95%CI (25%,59%)		2	1%, 95%CI (-0%,2%)	
	Overall	5	27%, 95%CI (12%,41%)		3	1%, 95%CI (0%,2%)	

Bold values indicates the classification of the adverse events.

Subgroup analyses of all grade AEs by different treatments suggested that no significant differences occurred in anemia (59 vs 45%, *p* = 0.31), thrombocytopenia (73 vs 64%, *p* = 0.19), decreased appetite (53 vs 33%, *p* = 0.14), diarrhea (45 vs 35%, *p* = 0.09), fatigue (69 vs 59%, *p* = 0.34) and neutropenia (36 vs 24%, *p* = 0.07). Subgroup analyses of all grade AEs by different treatments suggested that compared to SEL + DEX treatment, SEL + DEX + PIs treatment had a lower incidence of hyponatremia (39 vs 12%, *p* < 0.00001), nausea (72 vs 52%, *p* < 0.00001), vomiting (41 vs 23%, *p* < 0.0001) and weight loss (42 vs 17%, *p* = 0.03).

Subgroup analyses of grade≥3 AEs by different treatments revealed that no statistically significant subgroup differences were found between different treatment in terms of the incidence of decreased appetite (4 vs 3%, *p* = 0.86), thrombocytopenia (59 vs 52%, *p* = 0.41), diarrhea (6 vs 6%, *p* = 0.91), nausea (9 vs 7%, *p* = 0.52), neutropenia (22 vs 21%, *p* = 0.93) and vomiting (3 vs 4%, *p* = 0.82). Subgroup analyses of grade≥3 AEs by different treatments revealed that compared to SEL + DEX treatment, SEL + DEX + PIs treatment had a lower incidence of anemia (36 vs 16%, *p* = 0.02), fatigue (20 vs 13%, *p* = 0.04), hyponatremia (22 vs 5%, *p* < 0.0001).

## Disscussion

Multiple myeloma (MM) is a bone marrow-based malignant plasma-cell disorder without curative treatment, most patients will face the risk of relapse and develop refractory disease (Kumar et al., 2012; Gandhi et al., 2019; Kumar et al., 2019). It’s very meaningful to find more efficacious and less toxic treatment strategies for patients with relapsed/refractory multiple myeloma. Selinexor is an orally bioavailable, highly-selective, and slowly-reversible small molecule that binds to the Cys528 residue in the cargo-binding pocket of XPO1(12). We performed this meta-analysis to investigate the efficacy and safety of selinexor based treatments for multiple myeloma patients.

We included six studies, four studies were combined selinexor with dexamethasone and proteasome inhibitors regimens and two studies were combined selinexor with dexamethasone regimens. This review included a total of 276 MM patients treated with SEL + DEX + PIs treatment and 201 patients with SEL + DEX treatment. A random effects model was used when significant heterogeneity was observed in the analysis of efficacy and safety. Galbraith plots provides a visual impression of the amount of heterogeneity and indicates potential sources of heterogeneity. The results showed that one study (Grosicki et al., 2020) contributed mostly to publication bias in the pooled efficacy, and the reason for this phenomenon may be that this study provided the maximum number of enrolled patients. We also performed prespecified exploratory subgroup analyses by different treatments.

Our meta-analysis revealed that selinexor-based regimens could offer reasonable efficacy in patients with multiple myeloma. It worth noting that Selinexor exhibited a higher efficacy against MM resistant to previous therapies. The prior lines of therapy patients received ranged from 1 to 18, including iMiDs (lenalidomide, pomalidomide, thalidomide), PIs (bortezomib, Carfilzomib, Ixazomib), anti-CD38 monoclonal antibody (Daratumumab), Panobinostat, Alkylating Agent, antigen receptor-modified T cell therapy (CART), and ASCT. Patients with RRMM are heterogeneous, despite new treatment regimens improving outcomes, the prognosis remains particularly poor for heavily pretreated and/or multiple treatment-refractory patients (Usmani et al., 2016). Excessive nuclear export is an important factor in both the initiation and progression of cancer and is associated with resistance to chemotherapy (El-Tanani et al., 2016). Data from many preclinical studies confirm that selinexor effectively overcome different drug resistance, including bortezomib (Muz et al., 2017; Chanukuppa et al., 2019), anthracycline (Turner et al., 2013; Turner et al., 2016b), and so on. The results of subanalysis showed that SEL + DEX + PIs treatment could improve ORR, CBR, sCR, and VGPR compared to SEL + DEX treatment, while SEL + DEX treatment was associated with higher PDR (rate of progressive disease) and SDR (rate of stable disease) compared to SEL + DEX + PIs treatment. However, there was no difference in PR and MR between SEL + DEX + PIs treatment and SEL + DEX treatment. The combination of SEL and DEX has been approved by the FDA for patients with penta-refractory multiple myeloma. Due to the novel mechanism of action compared with other agents for MM, combining selinexor with other therapies is regarded as a promising therapeutic strategy. Our analysis revealed that the efficacy of SEL + DEX treatments could be enhanced by combinating with PIs. Furthermore, the pooled ORR (43%) in our analysis of SEL-based treatments was higher than that (39.1%) reported in a meta-analysis of bortezomib-based retreatment in relapsed/refractory myeloma (Knopf et al., 2014), revealing that the efficacy of SEL-based treatments was higher than bortezomib-based retreatment. Although the subgroup analysis of CR was not performed, in the RCT we included (Grosicki et al., 2020), the CR (7%) in the SEL + DEX + BTZ group was higher than the CR (4%) in the BTZ + DEX group. Considering that, selinexor-based regimen would be a good option for patients with R/R MM.

Although the recent approval of therapies, including next-generation PIs and immunomodulatory drugs (IMiDs), monoclonal antibodies (mAbs) and other new drug with novel mechanisms of action, has provided novel treatment options, MM remains an incurable disease, most patients will relapse and develop refractory disease to all available therapies (Kumar et al., 2012; Lonial et al., 2015; Kumar et al., 2019). Agents capable of harnessing the power of cellular immunity have long been sought, however, ASCT has remained the only proven therapy capable of long-term disease eradication through a graft-vs-myeloma effect (Nathwani et al., 2016). However, ASCT have a high treatment and transplant-related mortality rate (Giralt et al., 2015). It’s encouraging that the OS of patients with MM after ASCT has improved over time along with the introduction of new drugs for the treatment of MM (Shimazu et al., 2021). The development of other innovative immunotherapy approaches including monoclonal antibodies, chimeric antigen receptor T-cell therapy, checkpoint blockade, and novel vaccine therapies has led to a new era of immunotherapy in multiple myeloma (Nathwani et al., 2016). In addition to the combination with existing standard-of-care therapies like bortezomib, to explore the option of organically combining cellular immunotherapy with selinexor and transplantation for providing better treatment to multiple myeloma patients. Given the heterogeneity of RRMM, it’s critical to selected treatment plan more properly. Using the predictors of survival in patients such as early platelet engraftment and the administration of a CD34 ^+^ HPC count would be a rational choice (Aladağ Karakulak et al., 2020).

In our analysis, the most common AEs in all grade were thrombocytopenia, anemia, fatigue, nausea and decreased appetite. Besides, the most common AEs in grade≥3 were thrombocytopenia, anemia, neutropenia, and fatigue. Notably, hematological toxicities were the most frequent severe AEs. The pooled incidence of thrombocytopenia in all grade was 68%, 95%CI (60%,76%), and 54%, 95%CI (44%,65%) in grade≥3. The subanalysis showed no difference in the incidence of thrombocytopenia between SEL + DEX treatment and SEL + DEX + PIs treatment. Thrombocytopenia occurred in more than half of the patients, which was partly caused by selinexor because of its inhibition of thrombopoietin signaling in the differentiation of stem cells into megakaryocytes (Machlus et al., 2017). As thrombocytopenia was reversible and could be managed with dose interruptions, transfusion of blood products and thrombopoietin-receptor agonists, these patients rarely experienced severe thrombocytopenia (Machlus et al., 2017). Clinicians need to pay attention to the patients’ platelet status and adjust the medication regimen flexibly according to the patient’s platelet reduction. Bortezomib combined with dexamethasone was approved as a standard treatment in the United States for relapsed or refractory multiple myeloma. Peripheral neuropathy is the main dose-limiting toxicity caused by the standard treatment (Delforge et al., 2010; Cavaletti and Jakubowiak, 2010), In our included six studies, only two studies mentioned the incidence of peripheral neuropathy (Bahlis et al., 2018; Grosicki et al., 2020), other studies didn’t mention it because the incidence is too low. Both of the two studies used the selinexor + bortezomib + dexamethasone. However, the only one RCT study suggested that the triplet combination (SEL + DEX + PIs) was well tolerated with much lower rates of peripheral neuropathy compared to bortezomib + dexamethasone treatment in all grade (32 vs 47%) and grade≥3 (5 vs 9%) (Grosicki et al., 2020). Therefore, it seemed that the combination with selinexor was associated with lower rates and severity of bortezomib-induced peripheral neuropathy. The selinexor-based treatments have an improved side effect profile, while the SEL + DEX + PIs treatment has reduced levels of peripheral neuropathy. All patients involved in the meta-analysis received prophylactic use of antiemetics to mitigate gastrointestinal events. However, the all-oral Sid (selinexor, ixazomib, and low-dose dexamethasone) combination therapy for heavily pretreated patients (the patients had a median of five prior lines of therapy, ranged from 1 to 11 lines) with R/R MM resulted in frequent treatment delays and dose reductions owing to thrombocytopenia and gastrointestinal related toxicities, which lead to progression of disease (Salcedo et al., 2020). It suggested that heavily pretreated patients with R/R MM required careful consideration of using the combination therapy.

The subgroup analysis results suggested that SEL + DEX + PIs treatment was more effective and tolerated than SEL + DEX treatment for patients with MM. Preclinical data suggested that combining selinexor with proteasome inhibitors and dexamethasone had a mechanistic rationale (Turner et al., 2016a; Kashyap et al., 2016), as XPO1 is overexpressed in multiple myeloma and correlates with increased bone disease and shorter survival (Tai et al., 2014; Tiedemann et al., 2012), a genome-wide RNA interference screen identified XPO1 as an essential gene required for myeloma cell survival and proliferation (Schmidt et al., 2013). Furthermore, elevated levels of XPO1 is associated with the development of resistance to proteasome inhibitors (including bortezomib) (Chanukuppa et al., 2019) and immunomodulatory agents (Bhutani et al., 2017). Therefore, selinexor could inhibit the XPO1 and improve the resistance to PIs in R/R MM. Considering the relapsed/refractory status of the patient population enrolled in the meta-analysis, the efficacy of patients treated with selinexor-based treatments was satisfactory with tolerable AEs.

There are several inevitable limitations in our analysis. Firstly, most of the involved studies were single-armed studies without double-blinded randomized controlled trials. In addition, the dose of the drugs was different between individual trials, and some AEs were dose dependent. Finally, although the patients were all in advanced stages, the degree of multiple myeloma was dispersive, which might have aroused bias to the final analysis. No survival benefit was confirmed in our study due to the small sample size, the varied length of follow-up time and loss of data in some studies.

In conclusion, our meta-analysis further demonstrated that selinexor-based regimen was a novel and potent treatment option for patients with R/R multiple myeloma. The combination of selinexor with proteasome inhibitors and dexamethasone contributed to improve the efficacy and reduce the incidence of AEs than SEL + DEX treatment. Further studies are needed to explore the feasibility and efficacy of treatment for heavily pretreated patients with R/R MM. Combining XPO1 inhibitors with other effective antimyeloma agents and cellular immunotherapy was expected to be complementary to existing therapies for multiple myeloma, as well as for other neoplasms. Additionally, it is also important to focus on improving patients’ compliance, whether investigating new XPO1 inhibitors or new forms of combination.

## Data Availability

The original contributions presented in the study are included in the article/Supplementary Material, further inquiries can be directed to the corresponding author.
