# Supplementary material for: Safety and Efficacy Analysis of Selinexor-Based Treatment in Multiple Myeloma, a Meta-Analysis Based on Prospective Clinical Trials
Source: Front Pharmacol. 2021 Dec 3;12:758992. doi: 10.3389/fphar.2021.758992 (PMC8678413; doi:10.3389/fphar.2021.758992)
Supplement: Supplementary file 1 [file DataSheet1.docx]

Supplementary Material

# Supplementary Figures


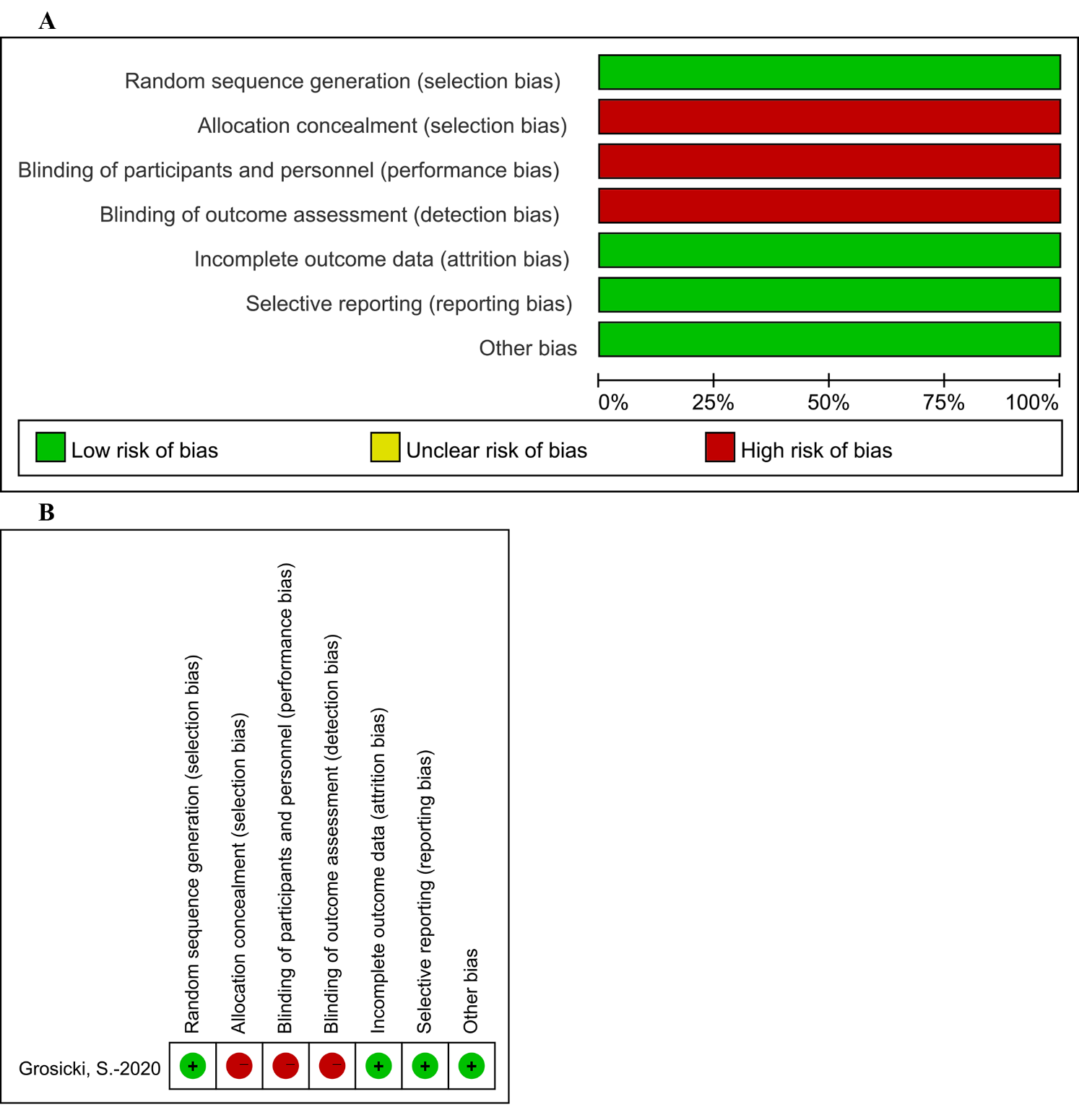


**Supplementary Figure 1.** Quality assessment of the included RCT study: (A) Risk of bias graph, (B)Risk of bias summary. +, Low risk of bias; –, high risk of bias; ?, unclear risk of bias


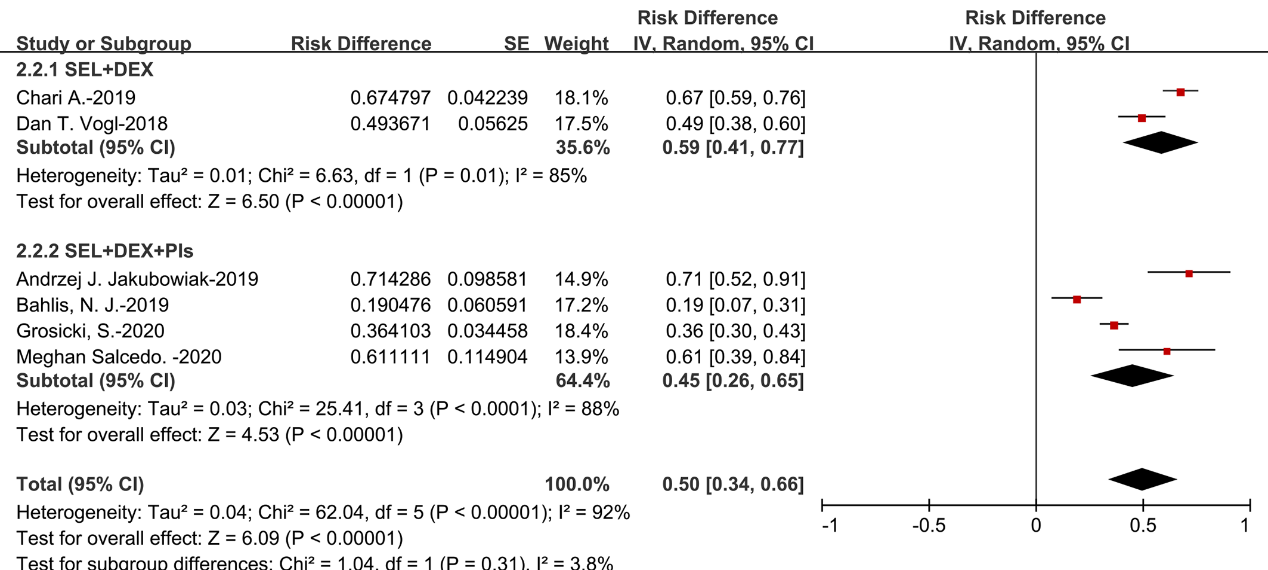


**Supplementary Figure 2.** The forest plot of pooled incidence of all grade anemia.


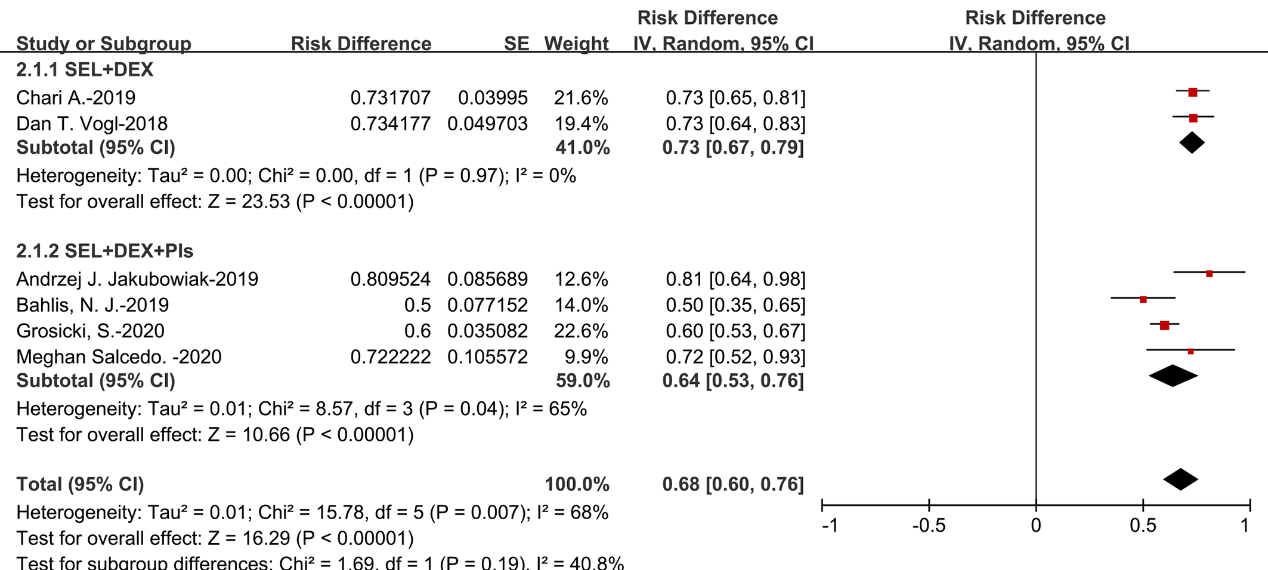


**Supplementary Figure 3.** The forest plot of pooled incidence of all grade thrombocytopenia.


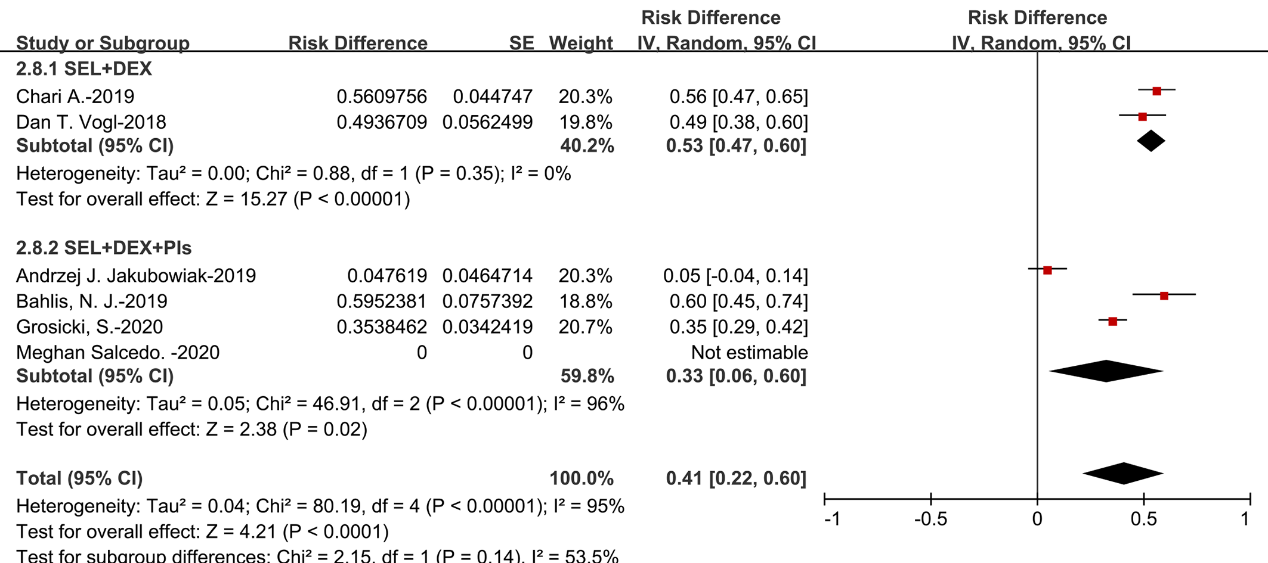


**Supplementary Figure 4.** The forest plot of pooled incidence of all grade decreased appetite.


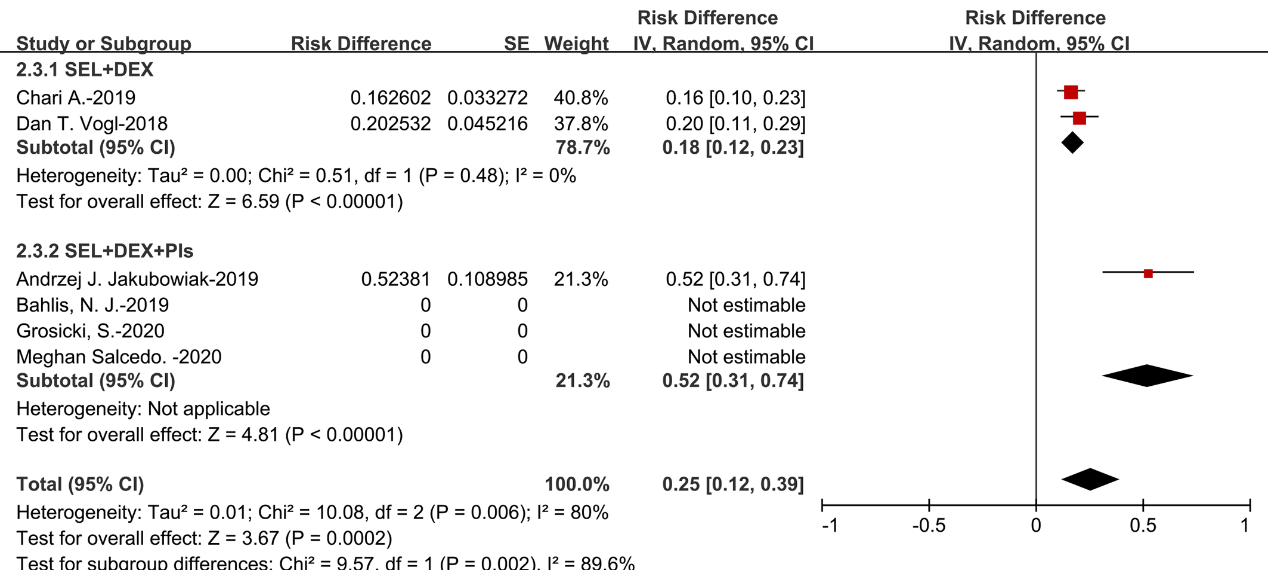


**Supplementary Figure 5.** The forest plot of pooled incidence of all grade lymphopenia.


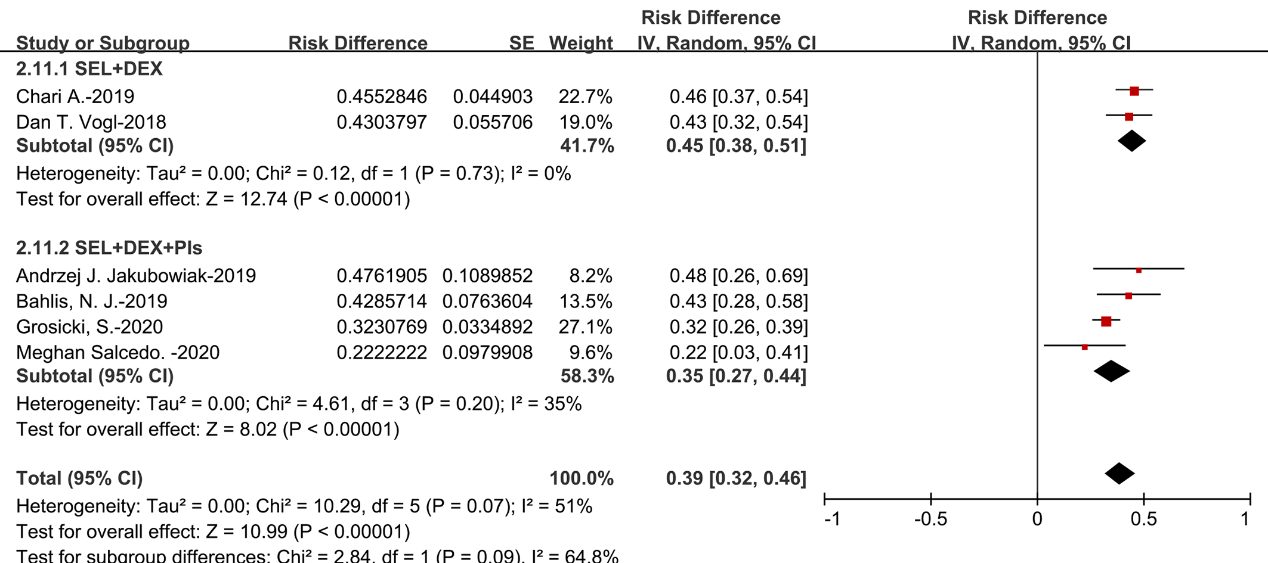


**Supplementary Figure 6.** The forest plot of pooled incidence of all grade diarrhoea.


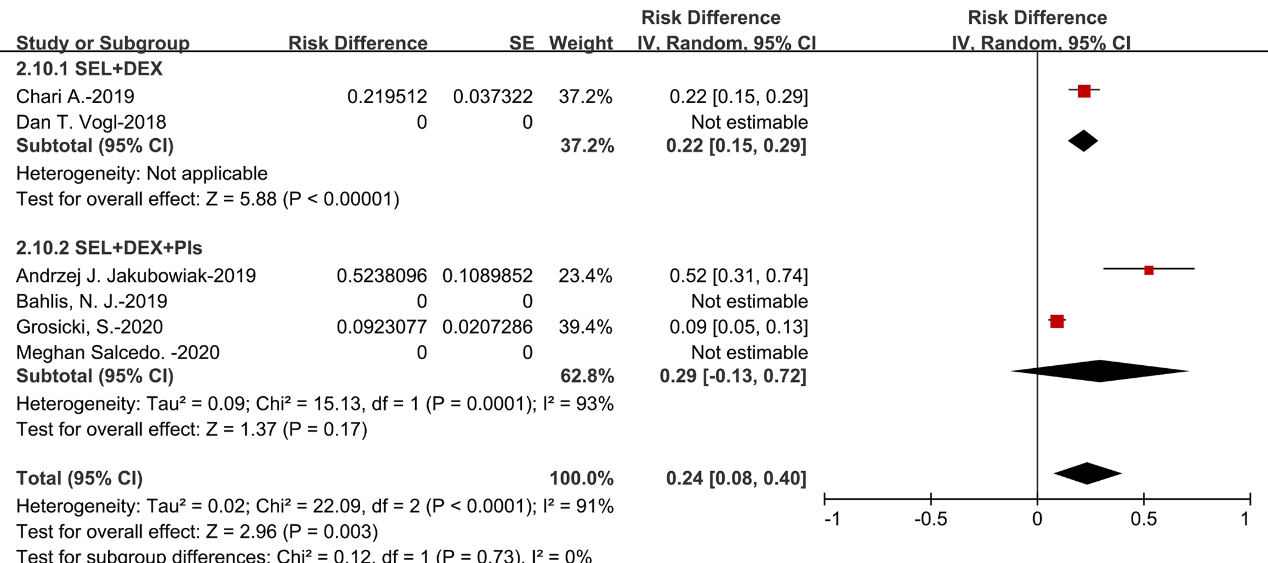


**Supplementary Figure 7.** The forest plot of pooled incidence of all grade dyspnoea.


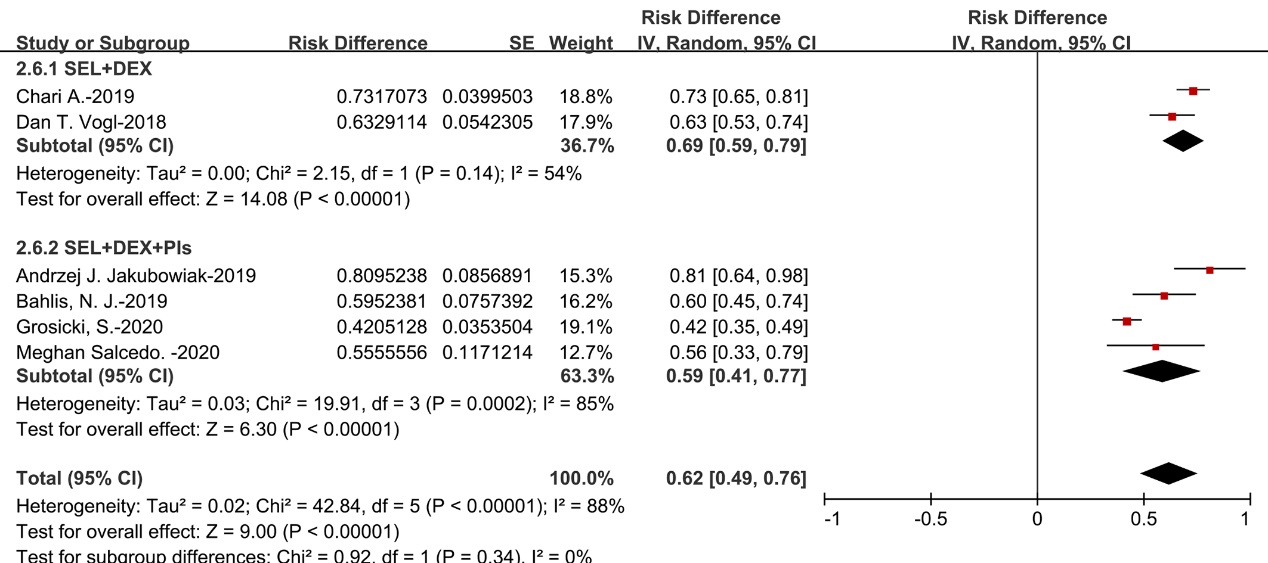


**Supplementary Figure 8.** The forest plot of pooled incidence of all grade fatigue.


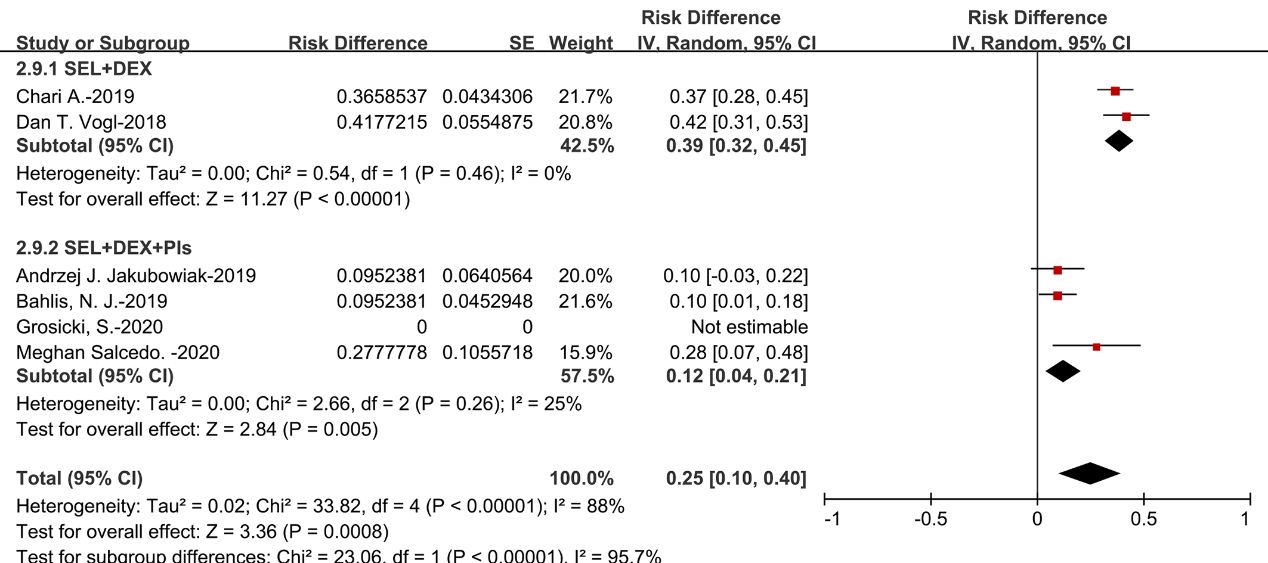


**Supplementary Figure 9.** The forest plot of pooled incidence of all grade hyponatremia.


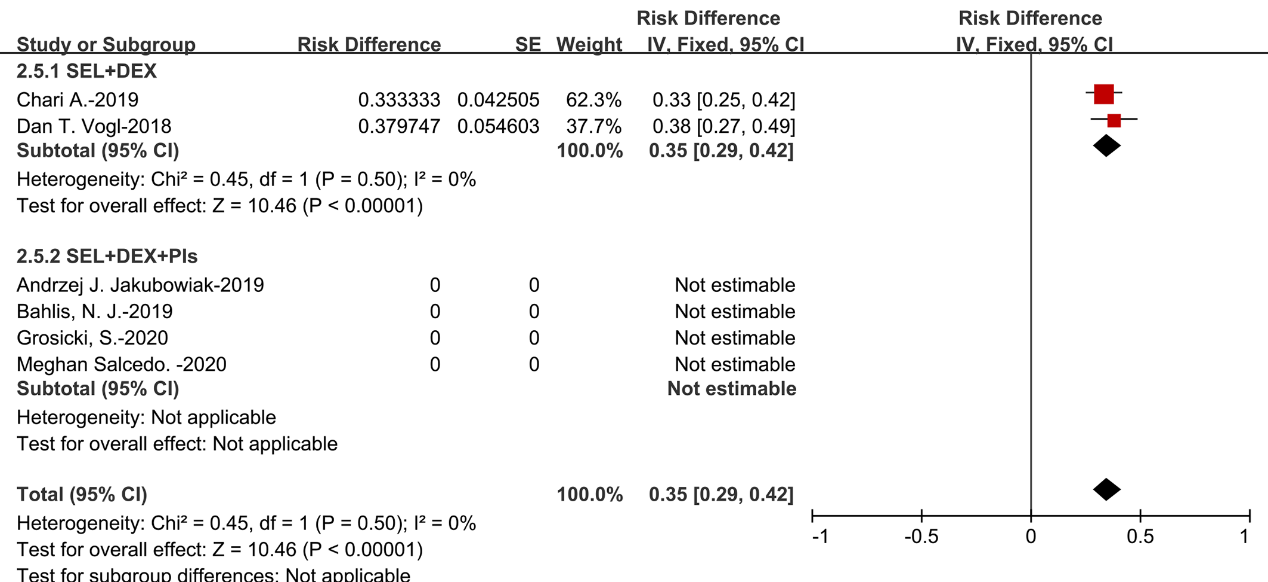


**Supplementary Figure 10.** The forest plot of pooled incidence of all grade leukopenia.


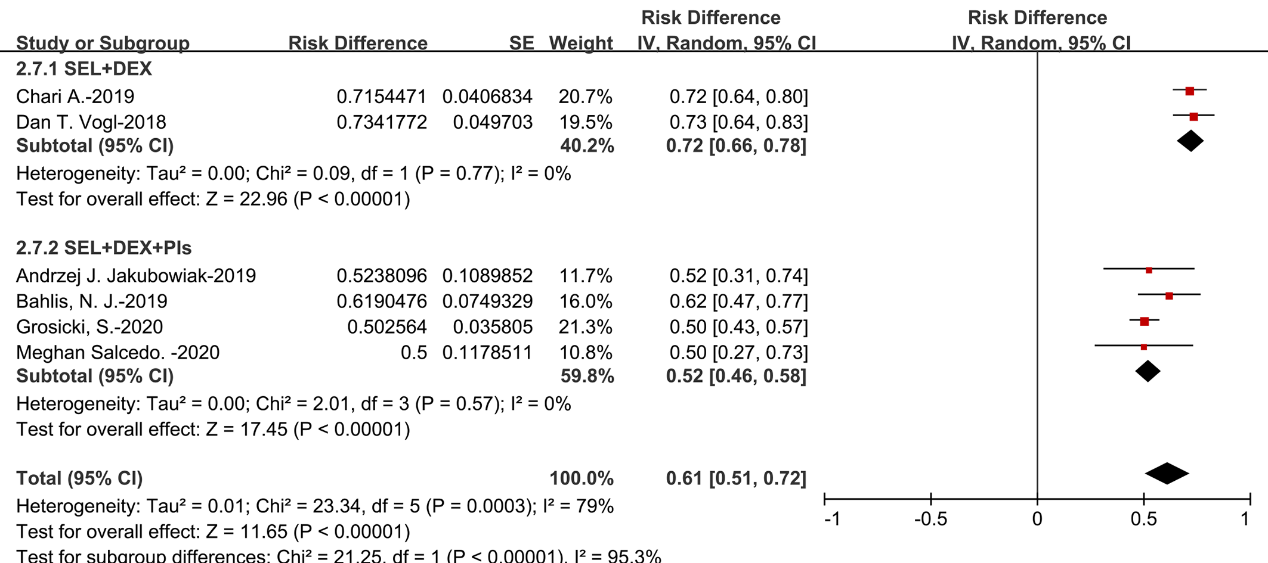


**Supplementary Figure 11.** The forest plot of pooled incidence of all grade nausea.


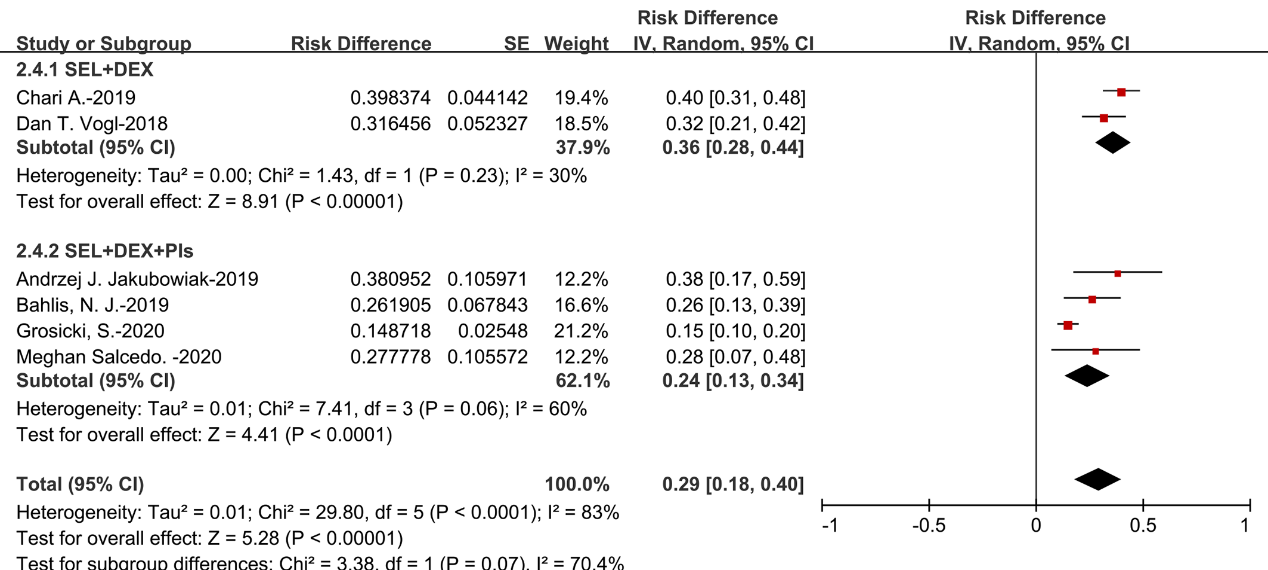


**Supplementary Figure 12.** The forest plot of pooled incidence of all grade neutropenia.


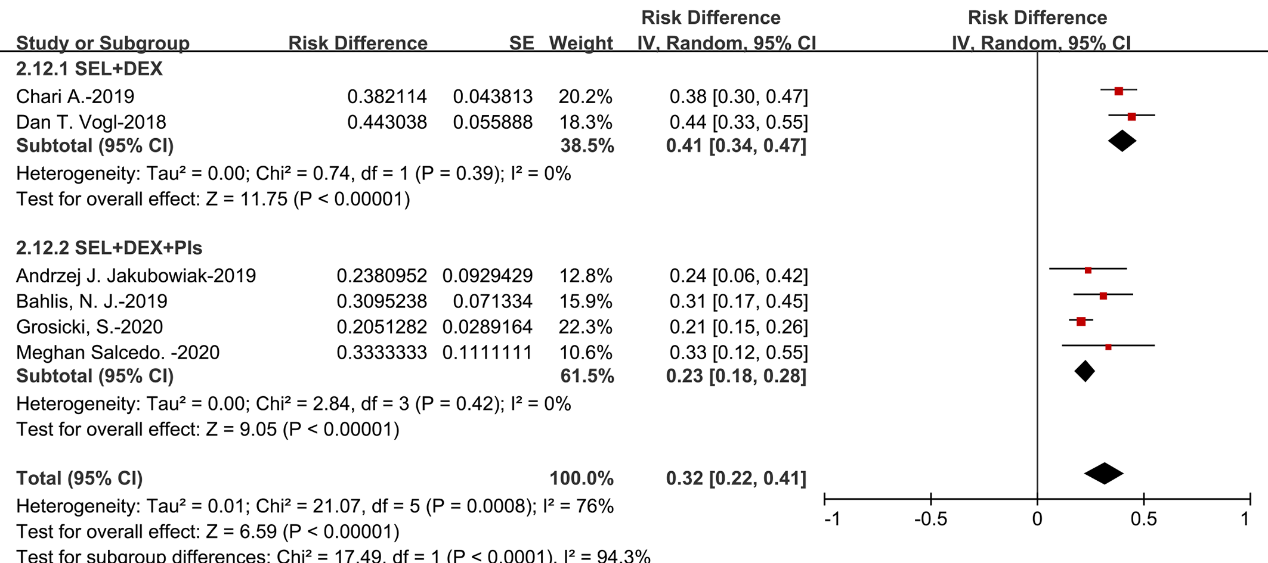


**Supplementary Figure 13.** The forest plot of pooled incidence of all grade vomiting.


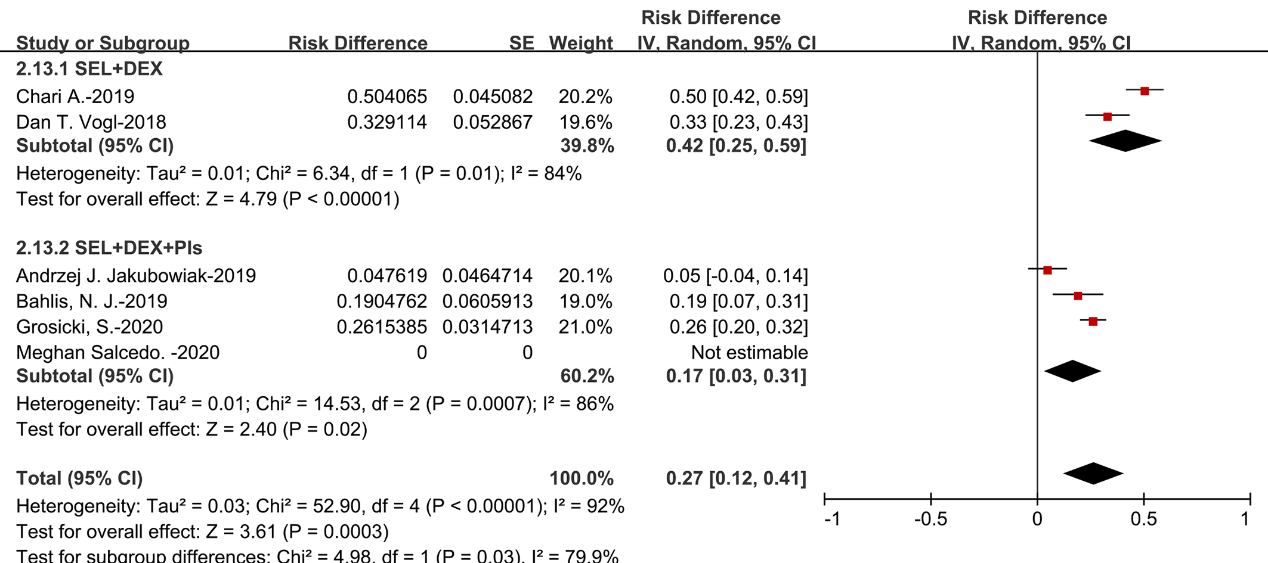


**Supplementary Figure 14.** The forest plot of pooled incidence of all grade weight loss.


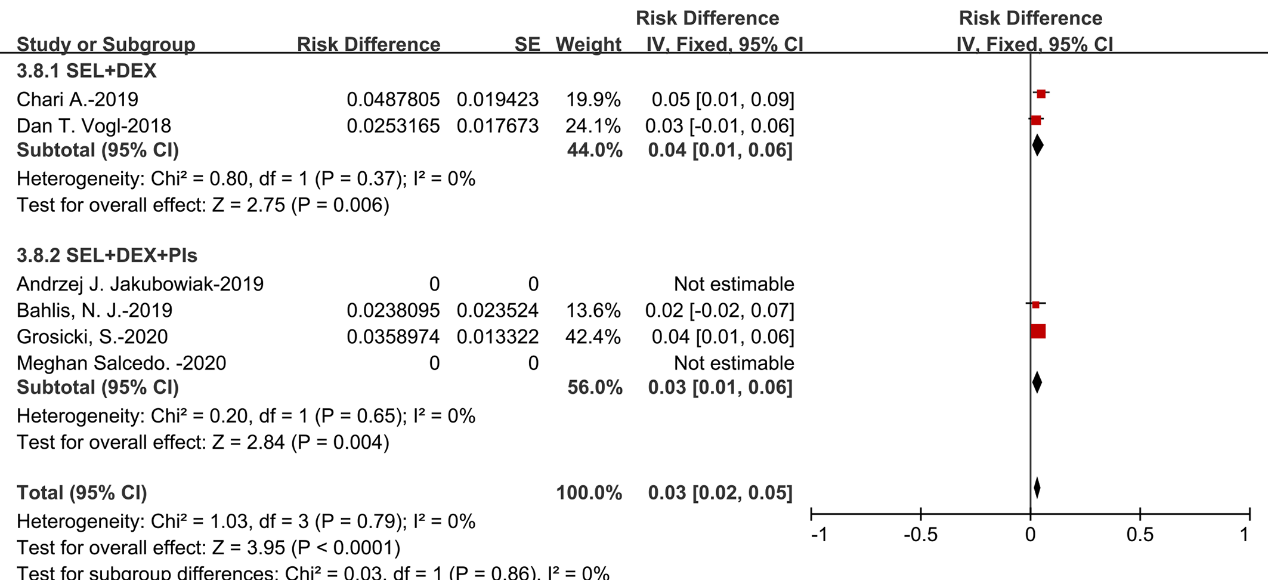


**Supplementary Figure 15.** The forest plot of pooled incidence of grade ≥3 decreased appetite.


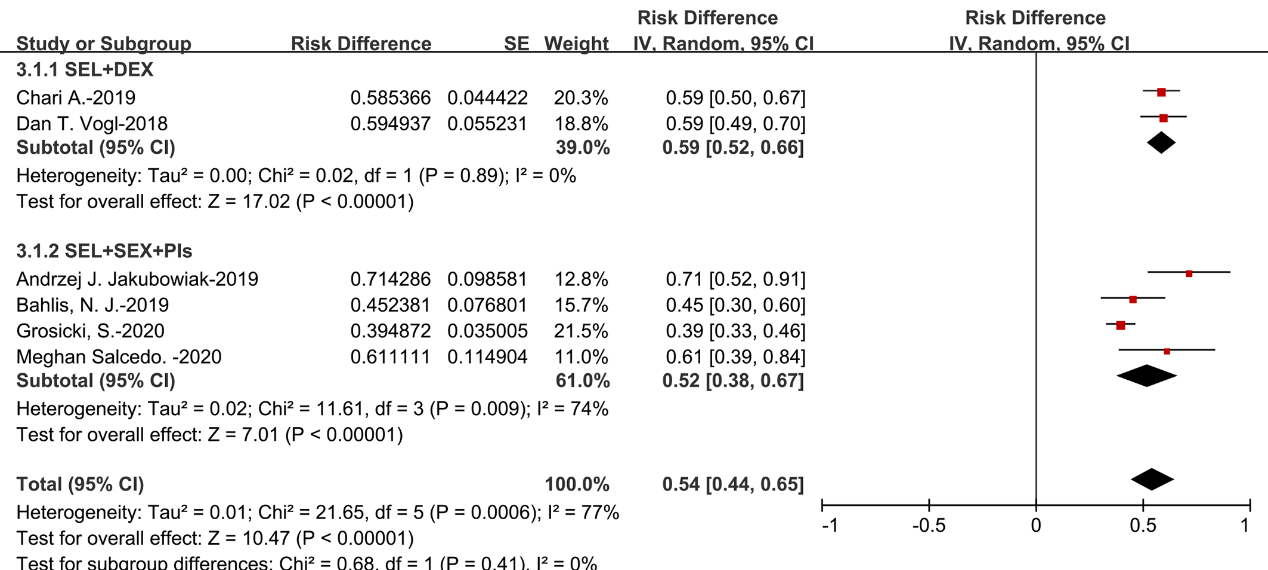


**Supplementary Figure 16.** The forest plot of pooled incidence of grade ≥3 thrombocytopenia.


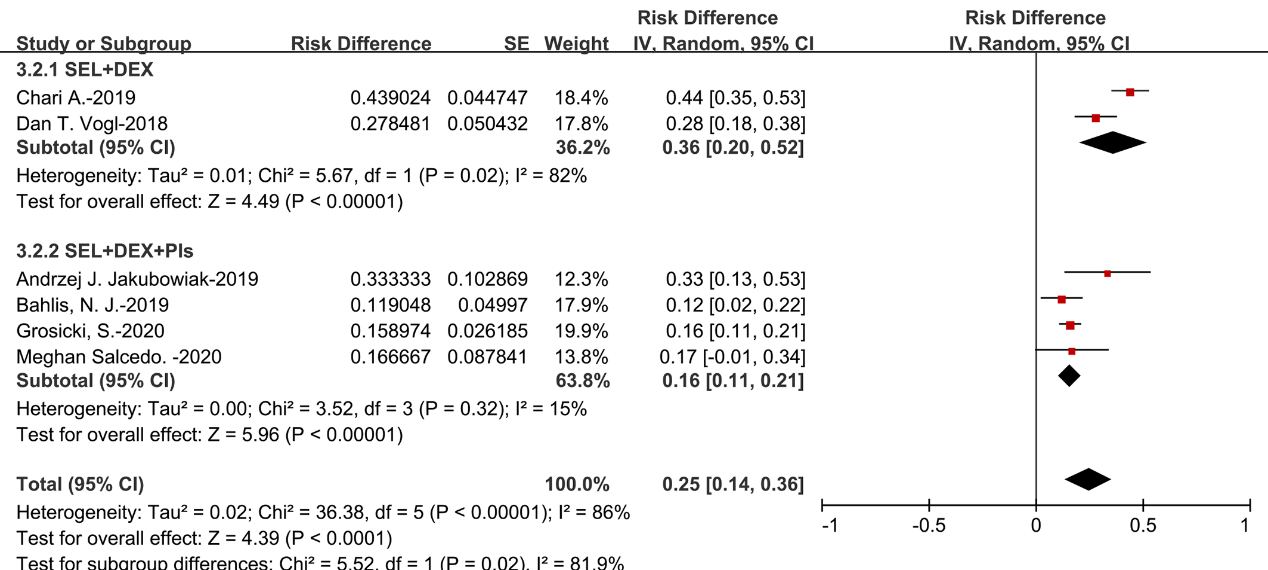


**Supplementary Figure 17.** The forest plot of pooled incidence of grade ≥3 anemia.


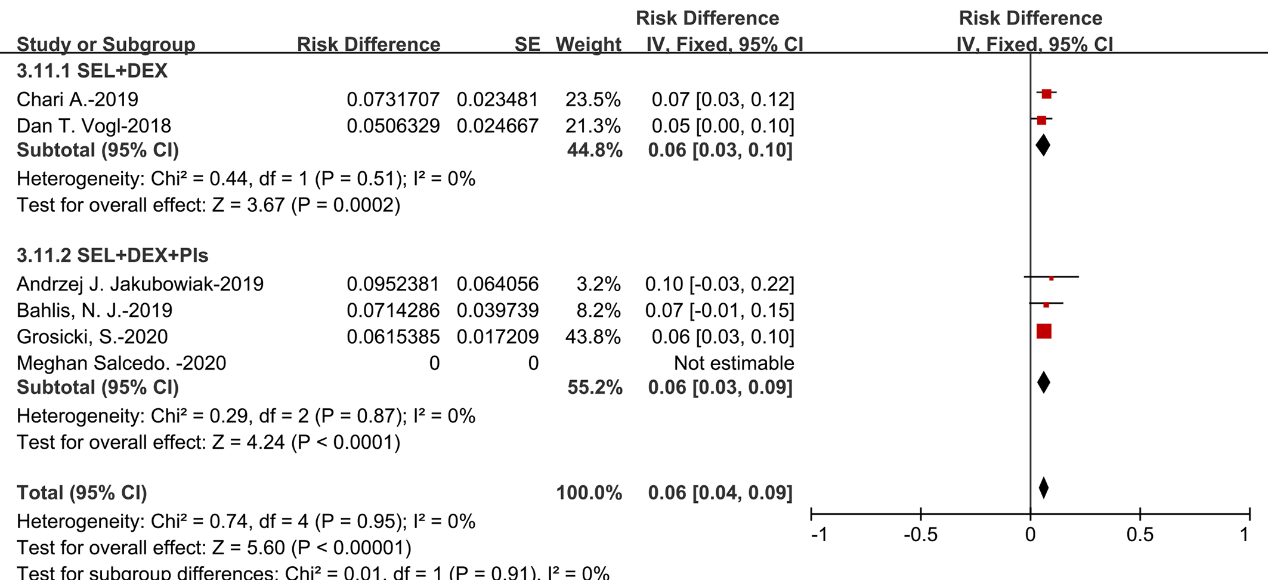


**Supplementary Figure 18.** The forest plot of pooled incidence of grade ≥3 diarrhoea.


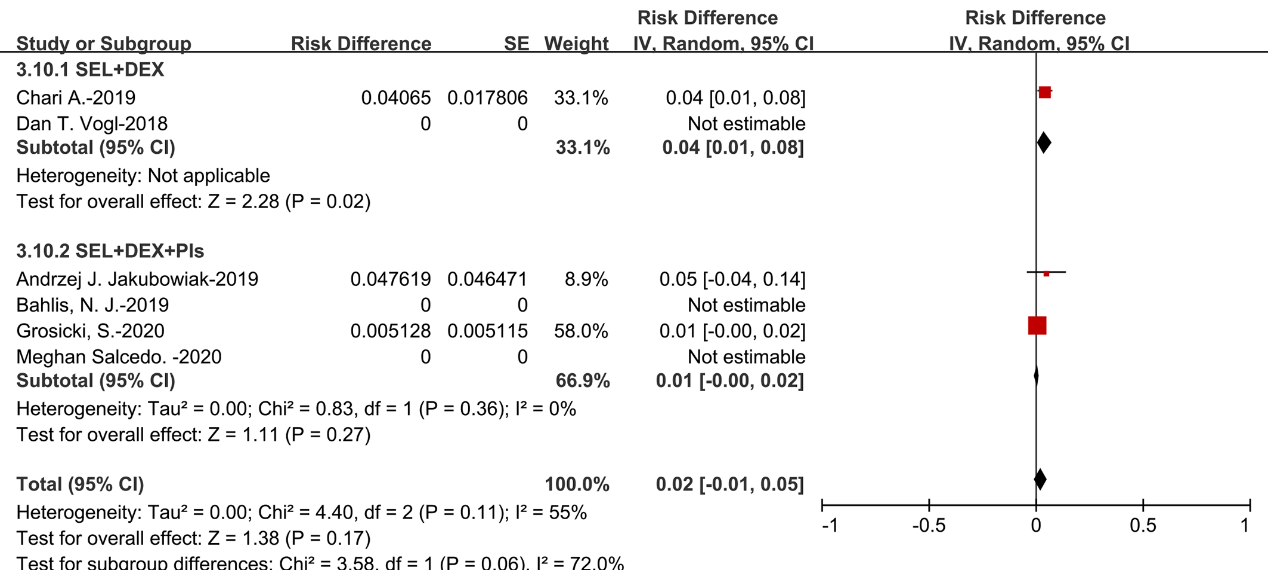


**Supplementary Figure 19.** The forest plot of pooled incidence of grade ≥3 dyspnoea.


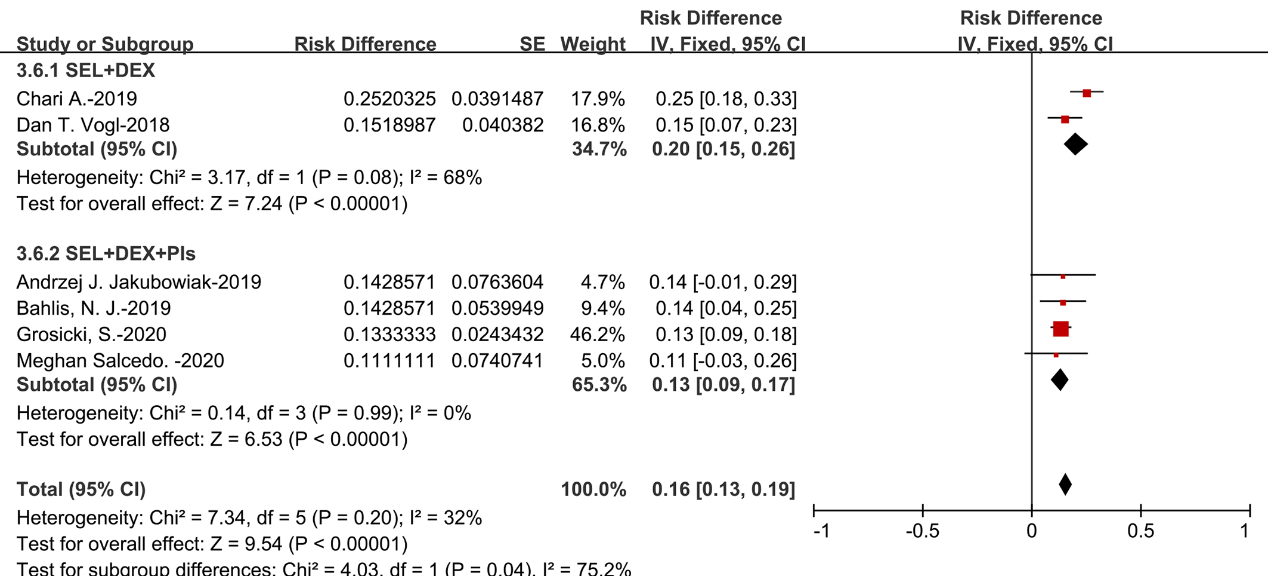


**Supplementary Figure 20.** The forest plot of pooled incidence of grade ≥3 fatigue.


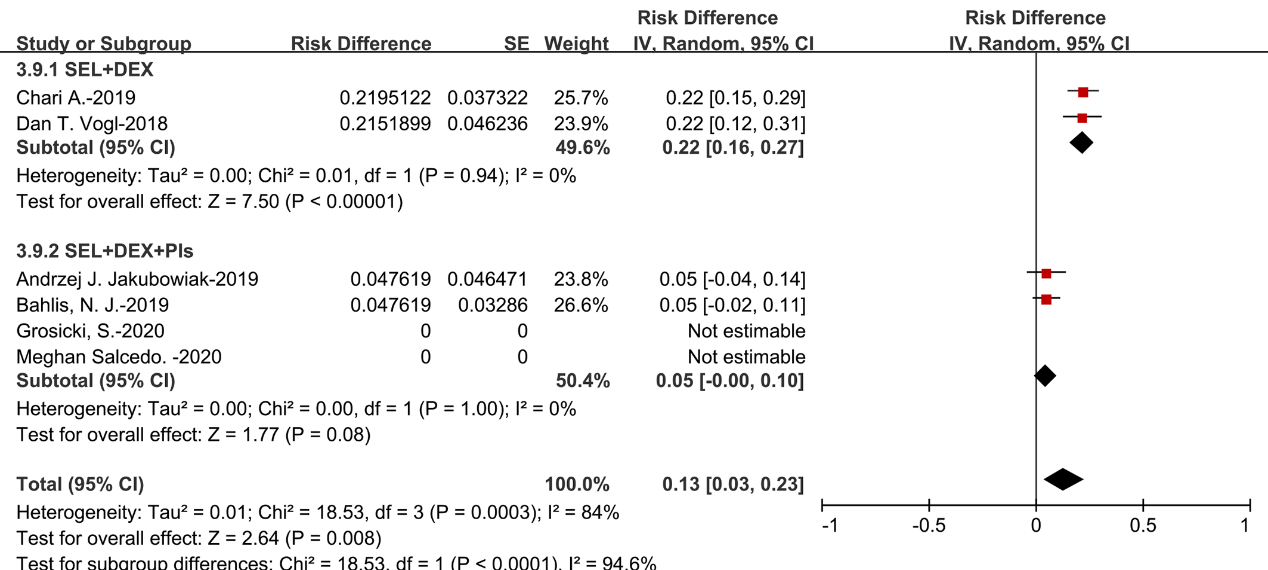


**Supplementary Figure 21.** The forest plot of pooled incidence of grade ≥3 hyponatremia.


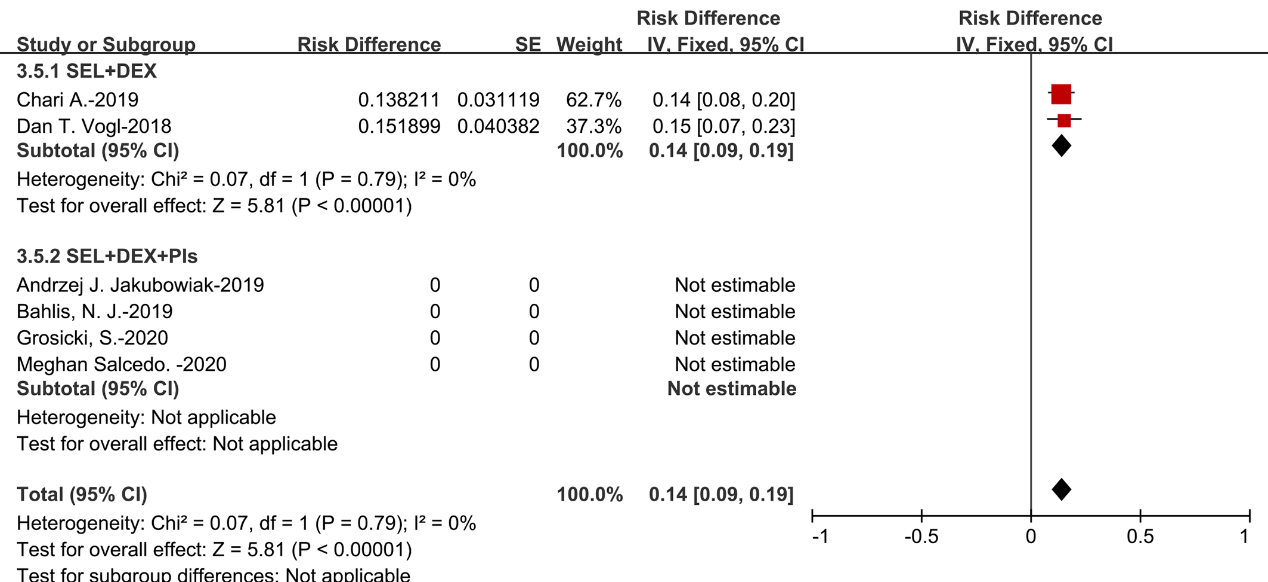


**Supplementary Figure 22.** The forest plot of pooled incidence of grade ≥3 leukopenia.


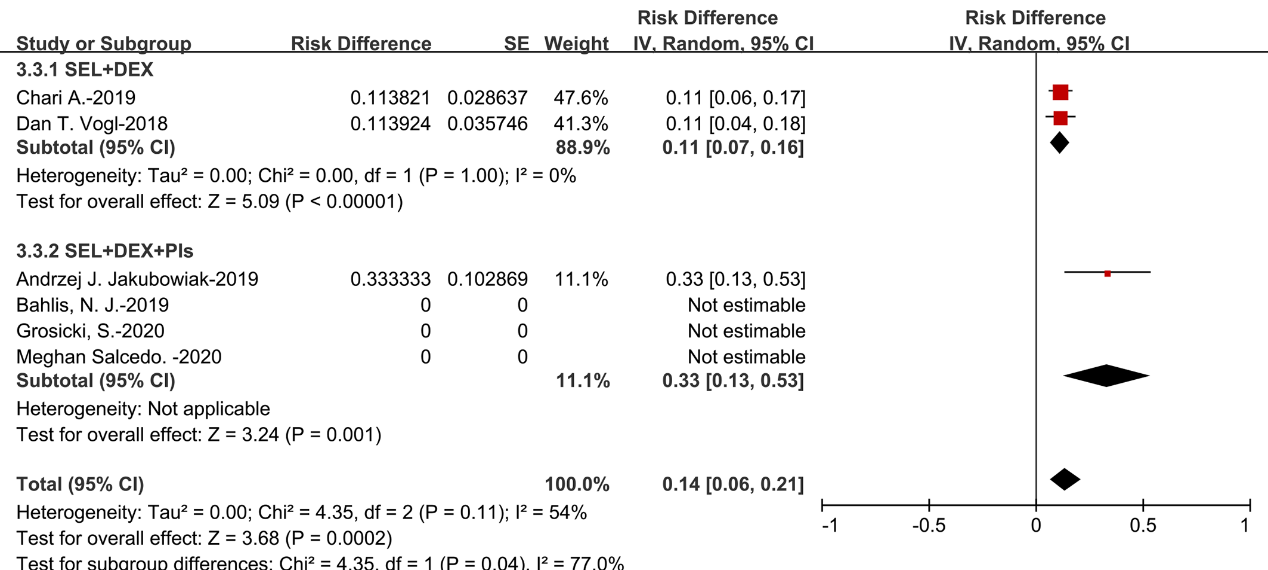


**Supplementary Figure 23.** The forest plot of pooled incidence of grade ≥3 lymphopenia.


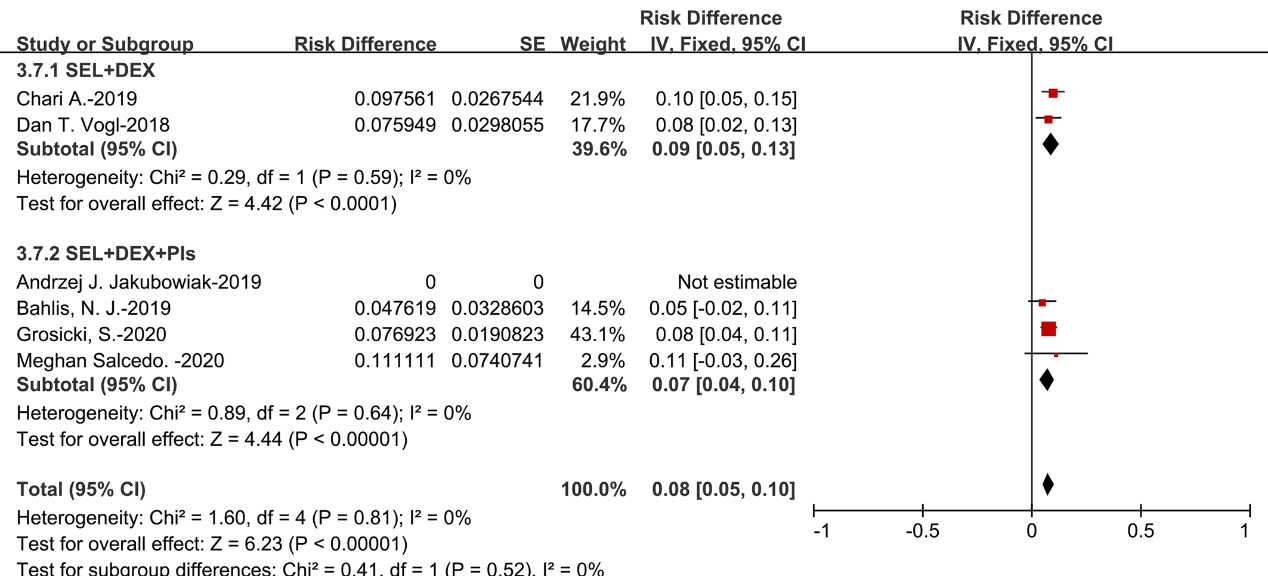


**Supplementary Figure 24.** The forest plot of pooled incidence of grade ≥3 nausea.


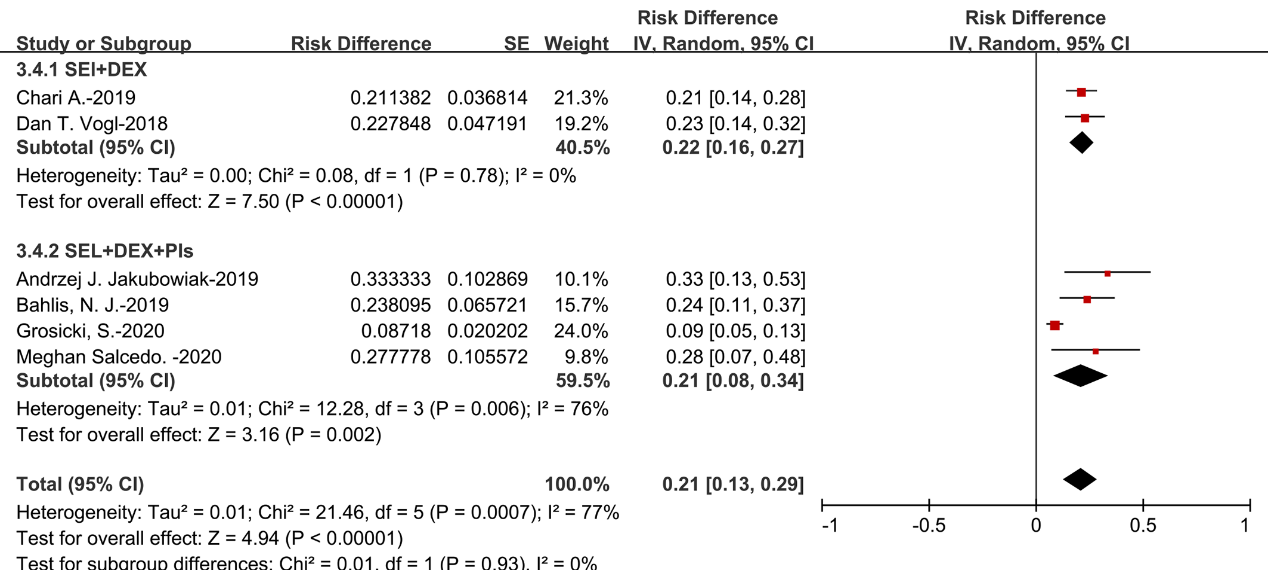


**Supplementary Figure 25.** The forest plot of pooled incidence of grade ≥3 neutropenia.


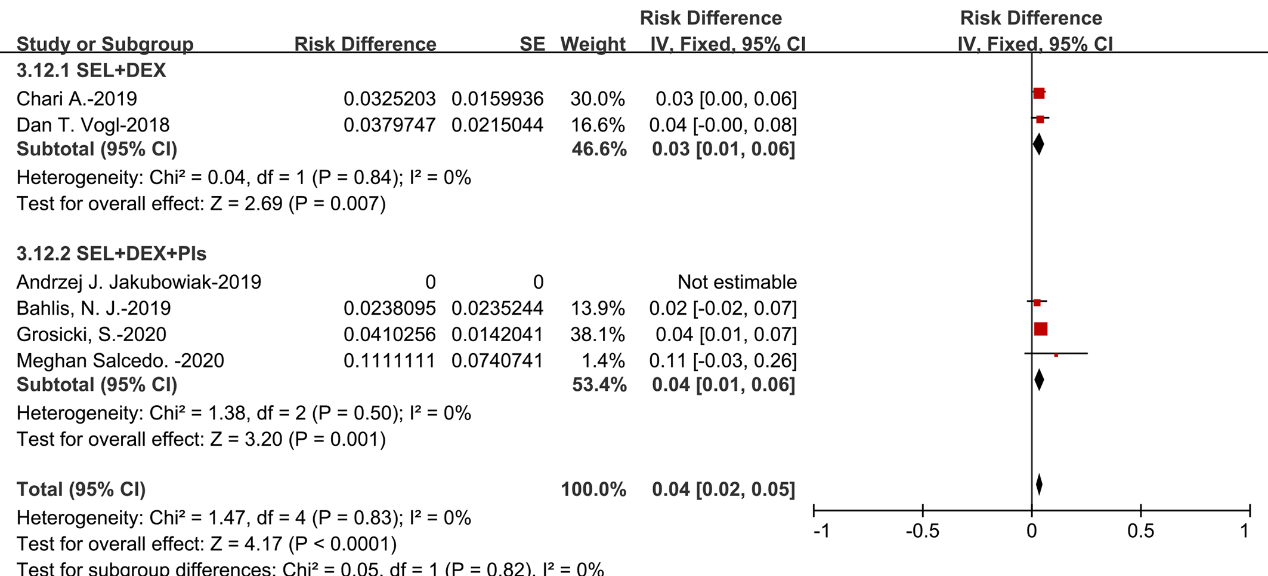


**Supplementary Figure 26.** The forest plot of pooled incidence of grade ≥3 vomiting.


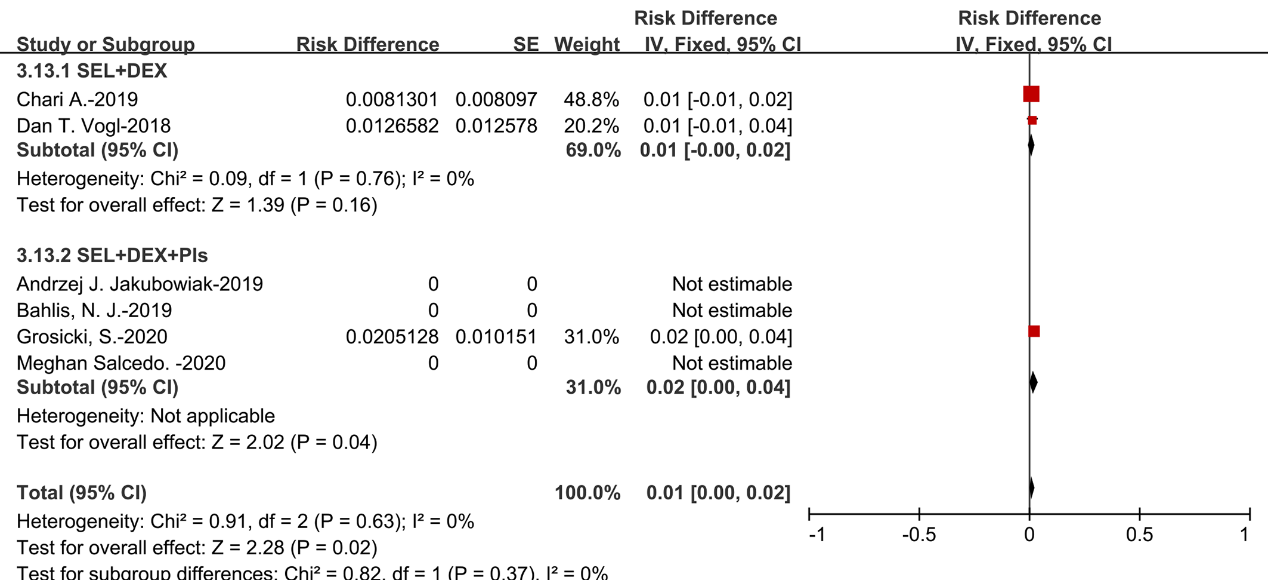


**Supplementary Figure 27.** The forest plot of pooled incidence of grade ≥3 weight loss.


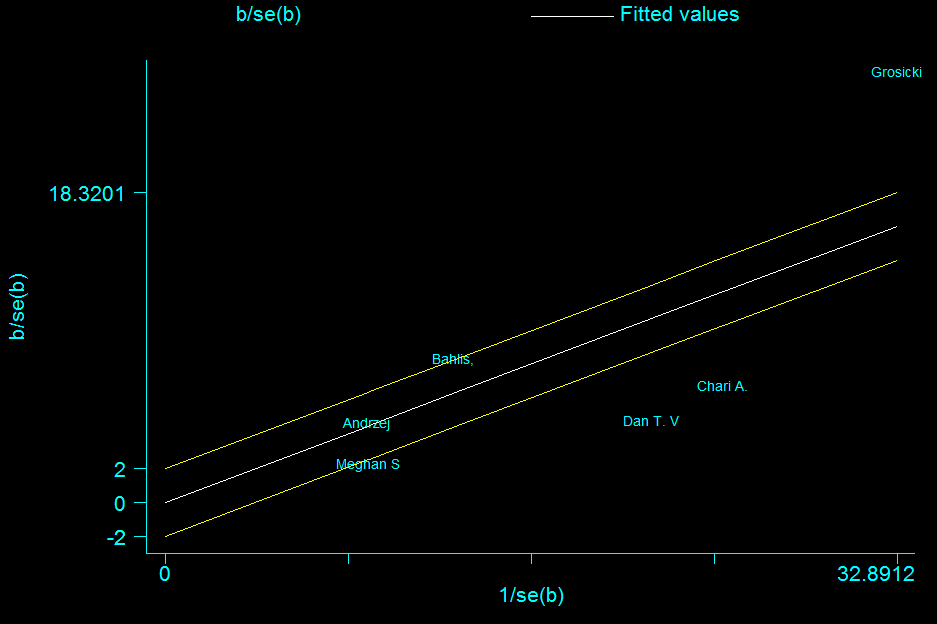


**Supplementary Figure 28.** The galbraith plot of ORR.


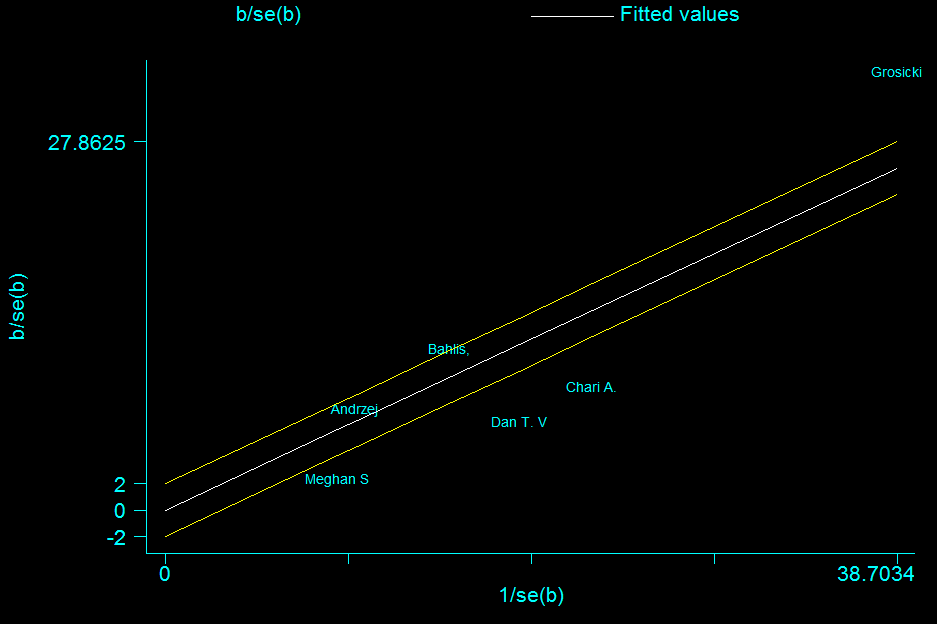


**Supplementary Figure 29.** The galbraith plot of CBR.


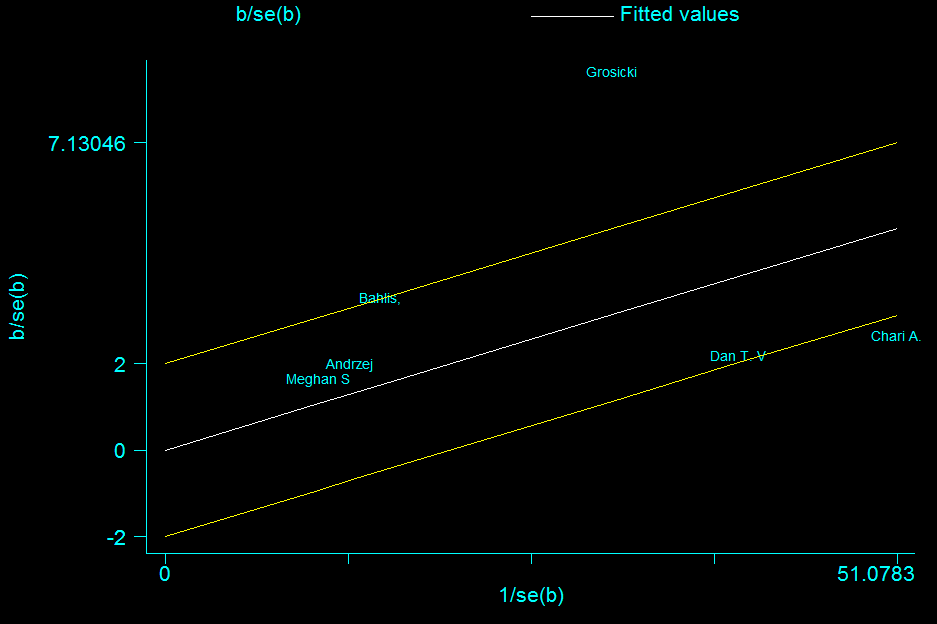


**Supplementary Figure 30.** The galbraith plot of VGPR.


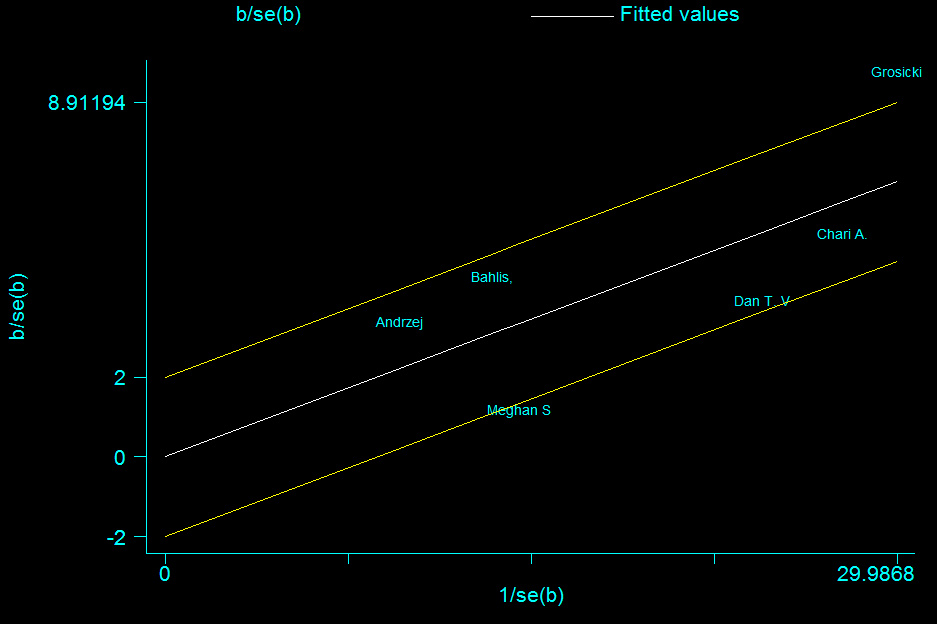


**Supplementary Figure 31.** The galbraith plot of PR.


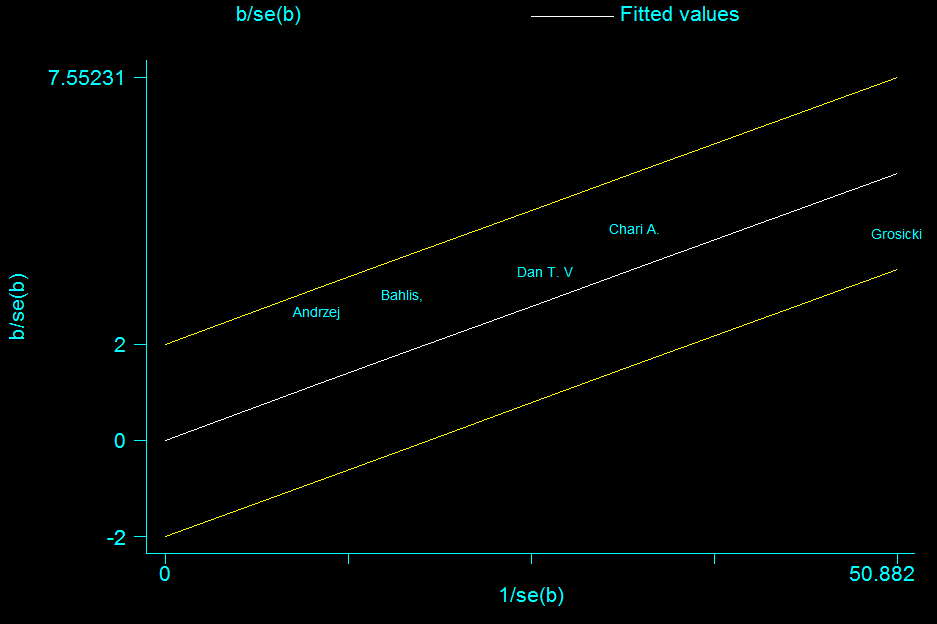


**Supplementary Figure 32.** The galbraith plot of MR.


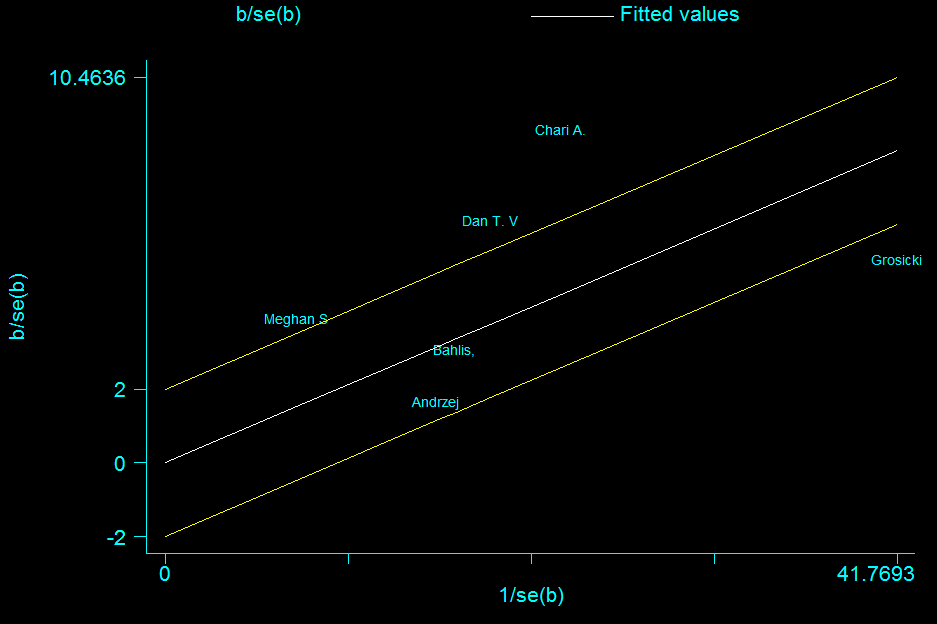


**Supplementary Figure 33.** The galbraith plot of SDR.


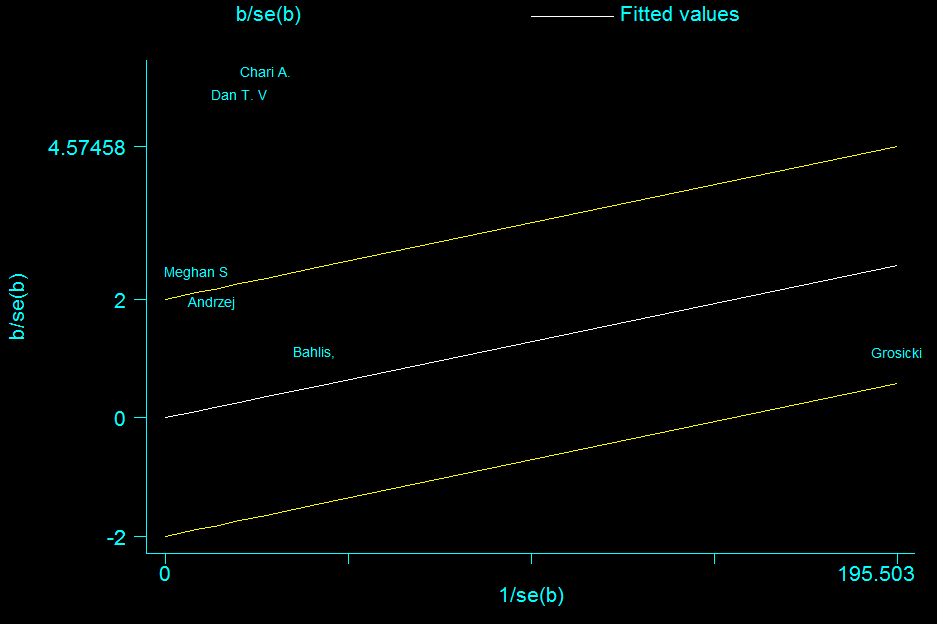


**Supplementary Figure 34.** The galbraith plot of PDR.
